# Caveolar Endocytosis Governs Nanoneedle Transfection

**DOI:** 10.1021/acsnano.5c11011

**Published:** 2026-02-06

**Authors:** Ningjia Sun, Cong Wang, Yikai Wang, William Edwards, Marija Dimitrievska, Yike Li, Nemanja Vasovic, Samuel McLennan, Hongting Zhu, Ermei Mäkilä, Jarno Salonen, Jiefei Shen, Qi Peng, Cristiano Scottà, Giovanna Lombardi, Ciro Chiappini

**Affiliations:** † Centre for Craniofacial and Regenerative Biology, 204287 King’s College London, London SE1 9RT, U.K.; ‡ Peter Gorer Department of Immunobiology, School of Immunology & Microbial Sciences, Faculty of Life Sciences & Medicine, 4616 King’s College London, London SE1 7EH, U.K.; § London Centre for Nanotechnology, 4616 King’s College London, London WC2R 2LS, U.K.; ∥ Wenzhou Eye Valley Innovation Centre, Eye Hospital, Wenzhou Medical University, Wenzhou 325024, China; ⊥ St John’s Institute of Dermatology, School of Basic & Medical Biosciences, 4616 King’s College London, London SE1 9RT, U.K.; # State Key Laboratory of Oral Diseases & National Centre for Stomatology & National Clinical Research Centre for Oral Diseases, West China Hospital of Stomatology, 12530 Sichuan University, Chengdu 610041, China; ∇ Department of Prosthodontics, West China Hospital of Stomatology, Sichuan University, Chengdu 610041, China; ○ Centre for Gene Therapy & Regenerative Medicine, King’s College London, London SE1 9RT, U.K.; 10 Department of Physics and Astronomy, University of Turku, Turku 20014, Finland; ◆ Department of Life Sciences, Centre for Inflammation Research and Translational Medicine, 3890 Brunel University London, London UB8 3PH, U.K.

**Keywords:** regulatory T cells, nanoneedle, Caveolin, endocytosis, nonviral transfection

## Abstract

Nanoneedles are emerging as a safe and scalable strategy for the genetic modification of primary human cells. However, a limited understanding of how interactions at the biointerface lead to functional gene expression continues to hinder clinical translation. While direct membrane penetration, permeabilization, and endocytosis have been proposed as intracellular delivery avenues, the mechanistic connection between delivery and successful transfection remains unclear. Here, we identify caveolae-mediated endocytosis, dependent on Caveolin-1, as a key mechanism enabling nanoneedle transfection. By selectively modulating Caveolin-1 expression in primary human regulatory T cells and MG63 cells and investigating endolysosomal processing, we show that although nucleic acids can be efficiently delivered in the absence of Caveolin-1, gene expression occurs only when caveolar endocytosis is present. These findings reveal a mechanistic basis and establish a broader design principle for nanoneedle transfection: interfacing must be accompanied by the engagement of permissive cellular trafficking pathways to achieve gene expression.

## Graphical abstract

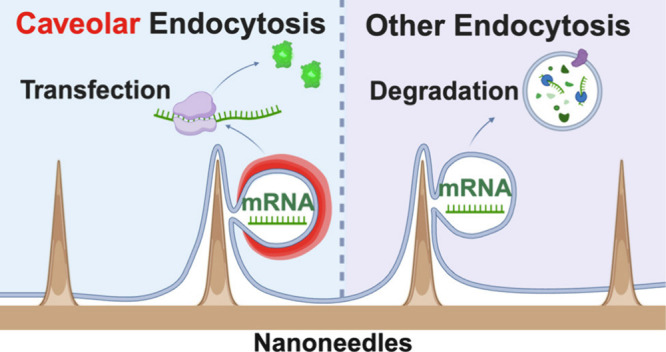

## 1 Introduction

Nanoneedles are high-aspect-ratio nanostructures that leverage biointerface interactions to enable nonperturbing intracellular access for efficient delivery, sensing, and bioelectronics. The cell-nanoneedle interface has been empirically optimized to transfect tissues^1–3^ and traditionally hard-to-transfect primary human cells,^4–7^ presenting a compelling alternative to viral delivery for advanced therapies. However, limited mechanistic insight into how these interactions lead to gene expression continues to hinder further optimization and clinical translation.

This limitation is particularly evident in lymphocytes, where achieving consistent and efficient transfection remains a significant challenge.^8–13^ Unlike adherent cells, lymphocytes possess distinct membrane properties and minimal basal endocytic activity, making a consistent transfection difficult. Attempts to enhance T-cell transfection via centrifugation or transfection reagent coatings yield only modest and inconsistent improvements.^14–16^ Among lymphocyte subsets, regulatory T cells (Tregs) present an even greater challenge,^17^ as their quiescent nature,^18,19^ distinct membrane composition,^20,21^ and heightened sensitivity to external stressors^22,23^ make them notably more resistant to genetic modification compared to conventional T cells.^24^ Importantly, nanoelectroporation—the integration of pulsed electrical stimulation with nanoneedles—appears essential for efficient T-cell transfection.^9,11,12,25,26^ These findings highlight the limitations of empirically optimizing nanoneedle transfection and underscore the need for a deeper understanding of the underlying mechanisms.

While membrane fluidization,^27–29^ direct penetration,^30–33^ and upregulated endocytosis^34–37^ have each been implicated in delivery from nanoneedles, their contributions to transfection remain undefined. Endocytosis, in particular, is an established route for transduction and transfection. Nanoneedles have been shown to upregulate clathrin-mediated endocytosis (CLME) and caveolae-mediated endocytosis (CavME).^35^ CLME and CavME contribute to the internalization of nucleic acids from cationic nanoparticles.^38–40^ Of these, only CavME typically supports transfection, since CLME often diverts cargo to lysosomal degradation.^41,42^ Caveolin-1 (CAV-1), a key driver of CavME, mediates viral entry for pathogens like Simian virus 40 (SV40) and Hepatitis B virus.^43–46^ The involvement of CavME in successful gene transfer indicates that this internalization pathway and CAV-1 expression might be pivotal for nanoneedle transfection.

Notably, primary lymphocytes show little or no CavME activity because low CAV-1 expression prevents caveolae formation.^47,48^ Although this may protect against viral infection,^49^ it could also limit nanoneedle-mediated gene transfer. Studying CAV-1 regulation and CavME in lymphocytes may therefore clarify the mechanisms underlying efficient nanoneedle transfection.

In this study, we directly tested whether CAV-1 expression is a critical determinant of nanoneedle transfection by comparing the transfection of primary human regulatory T-cells lacking CAV-1 (Treg −ve) and MG63 adherent cells that naturally express CAV-1 (MG63 +ve) alongside a CAV-1 knock-in variant of Tregs (Treg −ve) and a CAV-1 knockout variant of MG63 (MG63 −ve). We found that nanoneedles could efficiently deliver nucleic acids into Treg −ve, with up to 70% of cells receiving the payload. However, this did not result in successful transfection, as the delivered payload was trafficked through the endolysosomal system and rapidly degraded or excreted, failing to produce meaningful transgene expression. Instead, we achieved 54% transfection efficiency for MG63 +ve. However, the transfection efficiency dropped to 7% in MG63 −ve, while it increased to 35% in Treg +ve. These findings indicate that CAV-1 expression governs nanoneedle transfection through caveolae-mediated endocytosis.

## 2 Results and Discussion

### 2.1 Optimization of Nanoneedles-Tregs Interfacing

Centrifugation plays an essential role in optimizing nanoneedle interfacing with nonadherent cells like Tregs.^50–54^ First, we determined the ideal centrifugation duration by measuring the Treg metabolic activity (Figure 1a). CD4^+^CD25^+^ Tregs were isolated according to good manufacturing practice protocols^55^ published before, activated using anti-CD3/CD28 beads with 1000 IU/mL recombinant human IL-2 and 100 nM rapamycin. Centrifugation up to 60 min, whether on nanoneedles (nN RCF) or on a flat substrate (Flat RCF), did not significantly reduce the Treg metabolic activity. However, ATP luminescence increased in both nN RCF and Flat RCF groups after 60 min of centrifugation compared to shorter durations, indicating that extended centrifugation triggered increased ATP production, likely in response to cellular stress. Based on these findings, we selected 30 min as the optimal centrifugation duration, as it provided the most extended interfacing while not inducing observable metabolic stress.

**Figure 1:**
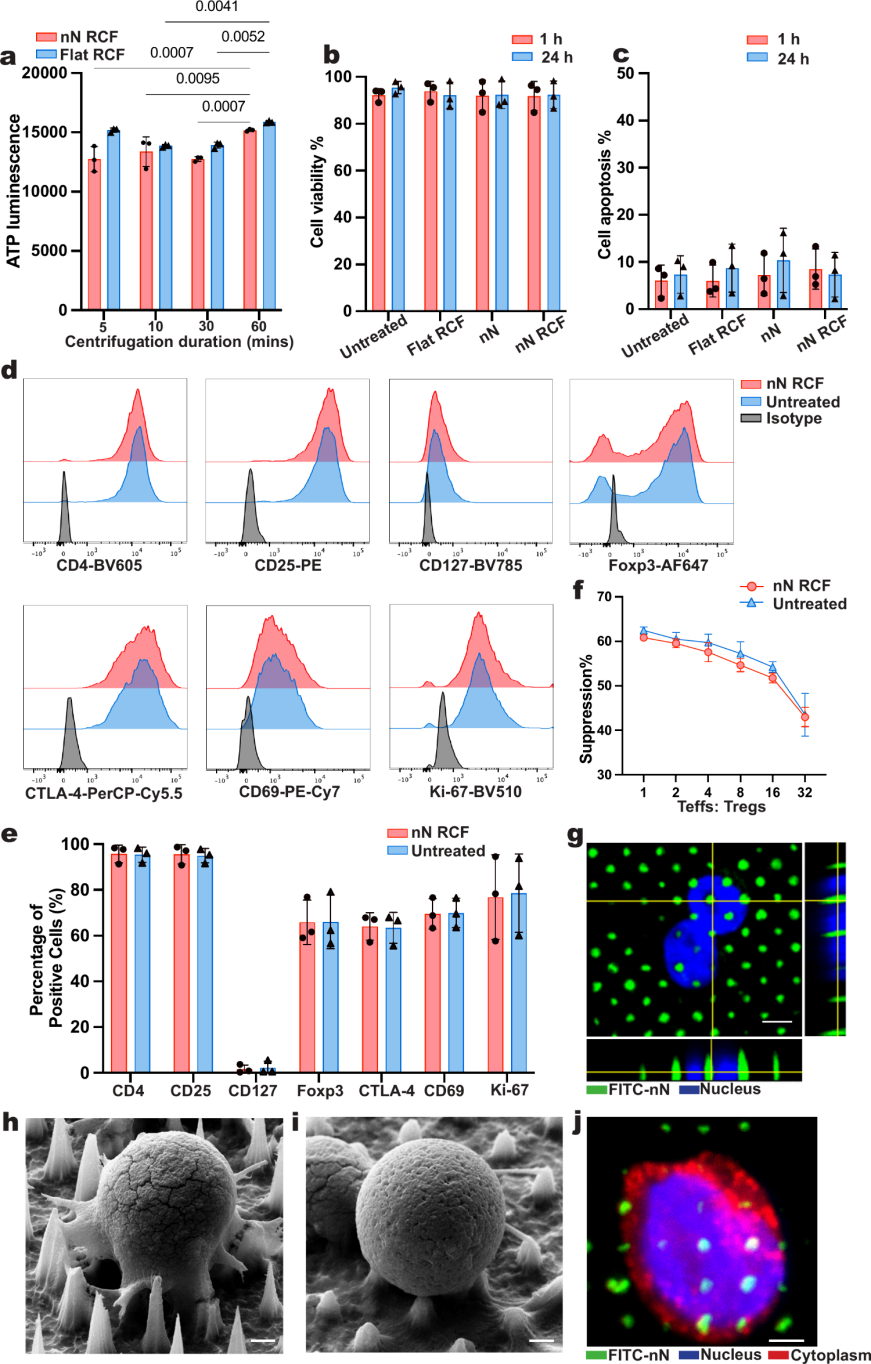
Nanoneedle interfacing with primary human regulatory T cells. (a) ATP concentration for Tregs as a function of nanoneedle centrifugation duration. Data presented as mean ± SD, *n* = 3 independent samples, two-way ANOVA followed by Tukey’s multiple comparison test. *p*-values are indicated above the bars. (b) Proportion of viable Tregs after 30 min of centrifugation on nanoneedles. Data presented as mean ± SD, *n* = 3 independent samples, two-way ANOVA followed by Tukey’s multiple comparison test. (c) Proportion of apoptotic Tregs following 30 min of centrifugation on nanoneedles. Data presented as mean ± SD, *n* = 3 independent samples, two-way ANOVA followed by Tukey’s multiple comparison test. (d) Representative flow cytometry histograms of characteristic Treg markers (CD4, CD25, CD127, and Foxp3), suppression marker CTLA-4, activation marker CD69, and proliferation marker *K*_i_-67. (e) Expression levels of the Tregs markers are shown in (d). Data presented as mean ± SD, *n* = 3 independent samples, two-way ANOVA, followed by Tukey’s multiple comparison test. (f) Tregs suppression of Teffs proliferation. Data presented as mean ± SD, *n* = 3 independent samples, two-way ANOVA, followed by Sidak’s multiple comparisons test. (g) Top view confocal microscopy image of the nucleus (DAPI – blue) of a Treg interacting with the nanoneedle array (FITC – green). Scale bar: 2 μm. (h,i) Scanning electron microscopy images of a Treg interacting with the nanoneedle substrate at (h) 1 h and (i) 24 h postseeding. Scale bar: 1 μm. (j) Top view confocal microscopy image of the cytoplasm (WGA – red) and nucleus (DAPI - blue) of a Treg on nanoneedles (FITC – green). Scale bar: 2 μm.

Next, we compared cell viability and apoptosis at 1 and 24 h between nN RCF, Flat RCF, and nanoneedle without centrifugation (nN). Across all groups, cell viability exceeded 90% and the apoptotic population was below 10% (Figure 1b,c, gating strategy Figure S1).

Having established that Tregs remain viable, we next assessed whether nN RCF affected Treg phenotype and function (Figure 1d,e). nN RCF Tregs retained their phenotype, with over 90% of cells being CD4^+^CD25^+^CD127^lo^. They maintained basal expression levels of the key functional markers CD69 (70% ± 7%), Foxp3 (66% ± 10%), CTLA-4 (64% ± 6%), and proliferation marker *K*_i_-67 (77% ± 19%). Finally, to determine whether nN RCF Tregs retained their suppressive ability, we cocultured them with CellTrace Violet (CTV)-labeled effector T cells (Teffs) in the presence of anti-CD3/CD28 beads for 5 days (Figure 1f). Across Treg-to-Teff ratios ranging from 1:1 to 1:32, the suppressive capacity of nN RCF Tregs was indistinguishable from that of untreated Tregs.

To visualize the nanoneedles-Tregs interface, we performed confocal and electron microscopy postcentrifugation (Figure 1g–j). Tregs were round with minimal cytoplasmic space, with the nuclear diameter being comparable to the overall cell diameter, of less than 10 μm. Their nuclei typically spread across three to five nanoneedles, appearing to wrap around the nanoneedles.

Taken together, these results demonstrate that nanoneedle interface effectively with primary human Tregs while preserving their viability, phenotype, and suppressive function.

### 2.2 Efficient Delivery Does Not Induce Transfection in Tregs

We first screened nanoneedle parameters to optimize Treg transfection. We compared the transfection efficiency of eGFP-mRNA for different surface derivatizations of silicon nanoneedles, including as-etched, oxidized, and thermally carbonized conditions. However, the transfection efficiency across all groups remained below 10%. Among them, the oxidized nanoneedles achieved the highest efficiency, averaging around 7%, whereas the thermally carbonized nanoneedles exhibited the lowest efficiency, with a statistically significant reduction compared to the oxidized condition (Figure S2a,b). Based on these results, we selected oxidized nanoneedles for all subsequent experiments. We then optimized nanoneedle height by fabricating short (2 μm), medium (3 μm), and long (6 μm) variants. SEM imaging confirmed that all nanoneedles had sharp, well-defined tips and uniform vertical alignment (Figure S2c–e). Quantitative analysis (Figure S2f) verified fabrication reproducibility, with distinct height distributions matching design parameters. Transfection efficiency for all height groups remained below 10% (Figure S2g,h). Previous studies indicate that 2–3 μm nanoneedles best interface with suspension immune cells, while longer structures (>3 μm) can reduce viability.^56^ Accordingly, the medium nanoneedle height was selected as optimal for subsequent experiments.

Importantly, the inherently difficult-to-transfect nature of Tregs was confirmed by the low lipofection efficiency observed in these cells (3% ± 2%), in contrast to the high efficiency in benchmark adherent MG63 cells (88% ± 2%) (Figure S3).

We quantified the delivery efficiency of Cy5-eGFP mRNA to establish its relationship with transfection, defined by the expression of the delivered gene. The nN RCF group achieved high delivery efficiency, with 70% ± 18% of Tregs showing Cy5 fluorescence, and a mean fluorescence intensity (MFI) of 31,795 ± 15,375 at 1 h post nanoinjection (Figure 2a,b). In contrast, nN yielded significantly lower delivery efficiency (27% ± 9%, MFI: 4362 ± 1114), and Flat RCF yielded only 7% ± 9% efficiency (MFI: 2602 ± 4129).

**Figure 2:**
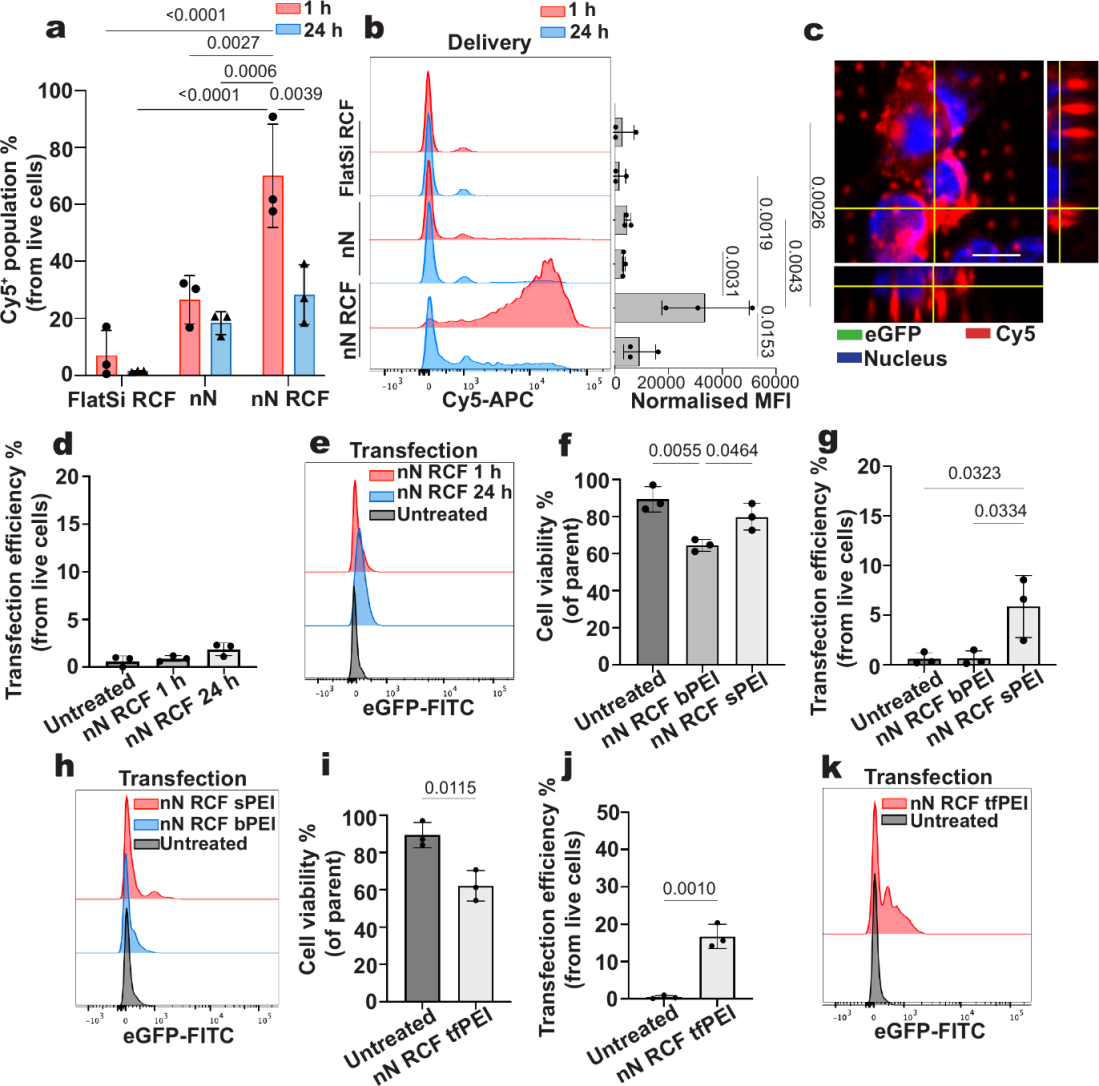
Nanoneedle delivery to Tregs does not induce transfection. (a) Efficiency of delivery into Tregs for Cy5-eGFP. Comparison between flat substrates with centrifugation (Flat RCF), nanoneedles without centrifugation (nN) and nanoneedles with centrifugation (nN RCF) at 1 and 24 h. Data presented as mean ± SD, *n* = 3 independent samples, two-way ANOVA followed by Tukey’s multiple comparisons test. *p*-values are indicated above the bars. (b) Representative flow cytometry histogram of the Cy5 fluorescence and Cy5 MFI value for all groups in (a). Data presented as mean ± SD, *n* = 3 independent samples, two-way ANOVA, followed by Tukey’s multiple comparisons test. *p*-values are indicated above the bars. (c) Top view confocal image showing Cy5^+^ Tregs interfaced with a nanoneedle array without detectable eGFP fluorescence. Cy5 is colored red, nucleus blue, and eGFP in green. Scale bar: 4 μm. (d) Transfection efficiency for nN RCF at 1 and 24 h compared to untransfected cells (Untreated). Data presented as mean ± SD, *n* = 3 independent samples, ordinary one-way ANOVA, followed by Tukey’s multiple comparisons test. (e) Representative flow cytometry histogram of the eGFP fluorescence for groups presented in (d). (f) Cell viability for untreated, nN RCF treated with branched PEI (nN RCF bPEI), and silane PEI (nN RCF sPEI). Data presented as mean ± SD, *n* = 3 independent samples, ordinary one-way ANOVA, followed by Tukey’s multiple comparisons test. *p*-values are indicated above the bars. (g) Transfection efficiency for untreated, nN RCF bPEI, and nN RCF sPEI at 24 h post nanoinjection. Data presented as mean ± SD, *n* = 3 independent samples, ordinary one-way ANOVA, followed by Tukey’s multiple comparisons test. *p*-values are indicated above the bars. (h) Representative flow cytometry histogram of eGFP fluorescence for groups presented in (g). (i) Cell viability for untreated and nN RCF tfPEI at 24 h post nanoinjection. Data presented as mean ± SD, *n* = 3 independent samples, unpaired *t* test, and *p*-value is indicated above the bars. (j) Transfection efficiency for untreated and nN RCF tfPEI at 24 h post nanoinjection. Data presented as mean ± SD, *n* = 3 independent samples, unpaired *t* test, *p*-value is indicated above the bars. (k) Representative flow cytometry histogram showing eGFP fluorescence for groups presented in (j).

Although a large proportion of Tregs initially received the payload, as indicated by Cy5 fluorescence, the proportion of Cy5-positive cells decreased significantly to 28% ± 11% after 24 h. Furthermore, following delivery, negligible eGFP expression was observed, as confirmed by confocal microscopy (Figure 2c) and flow cytometry (Figure 2d,e). These results suggest that, although nanoneedles facilitated the delivery into a large proportion of Tregs, the payload failed to present functionally in the cytosol, possibly through endolysosomal trafficking, leading to a combination of degradation and/or excretion.

To enhance endosomal escape, we functionalized nanoneedles with adsorbed branched polyethylenimine (bPEI), a well-established cationic transfection reagent that promotes uptake and disrupts endosomal membranes through the proton sponge effect. bPEI functionalization (nN RCF bPEI) significantly compromised Treg viability, reducing it to 64% ± 3% and still failed to achieve detectable transfection (Figure 2f–h). This toxicity likely stemmed from the high density of positive charges in PEI, which can disrupt the cell membrane integrity through strong interactions with negatively charged cellular components.

A recent study in CD8+ T cells reported that grafting PEI on nanoneedles enhanced transfection efficiency while reducing cytotoxicity compared to adsorbed PEI.^14^ Thus, we adopted this approach to test whether similar benefits could be observed in our platform by grafting Silane-PEG-NHS conjugated PEI (sPEI) on nanoneedles. This modification (nN RCF sPEI) improved cell viability to 80% ± 7% and resulted in a modest transfection efficiency of 6% ± 3% (Figure 2f–h).

Given the limited efficacy of PEI alone, we next explored whether combining it with specific endocytic uptake could further improve the transfection. To this end, we coated nanoneedles with a transferrin-PEI (tfPEI) conjugate, leveraging transferrin’s known uptake via CLME. This dual-functional approach aimed to enhance both cellular internalization and endosomal escape. This strategy further improved transfection efficiency to 17% ± 3% but with diminished cell viability (62% ± 8%) (Figure 2i–k).

Together, these results highlight the challenges of achieving efficient nanoneedle transfection in Tregs. While enhanced uptake and endosomal escape strategies modestly improve transfection, the reliance on CLME appears to direct the payload toward lysosomal degradation or excretion, limiting gene expression. Overcoming these barriers requires a deeper understanding of the internalization pathways in order to develop strategies to reroute or bypass degradative trafficking. Without such advancements, nanoneedles remain unsuitable for robust genetic engineering of Tregs.

### 2.3 Endolysosomal Trafficking of mRNA

To determine whether endolysosomal trafficking limited transgene expression despite efficient delivery, we investigated mRNA processing.

We quantified the levels of full-length intracellular eGFP mRNA in Tregs at 1, 16, and 48 h after nanoneedle transfection (Figure S4). Full-length eGFP mRNA was readily detectable at 1 h, confirming rapid uptake and delivery into the cells. However, by 16 and 48 h, the relative abundance decreased by approximately 10-fold compared with the 1 h level, indicating that the delivered eGFP mRNA underwent progressive degradation or was recycled out of the cells over time, suggesting lysosomal trafficking.

Indeed, confocal microscopy revealed a time-dependent increase in the colocalization of Cy5-mRNA with lysosomal compartments (Figure 3a). Quantitative analysis confirmed this trend, showing that colocalization observed at 3 h further increased by 8 h, as indicated by the increase in both Manders’ coefficient and Pearson’s correlation (Figure 3b,c). By 24 h, Cy5-mRNA fluorescence was largely diminished, consistent with the progressive lysosomal degradation of the cargo (Figure 3a).

**Figure 3:**
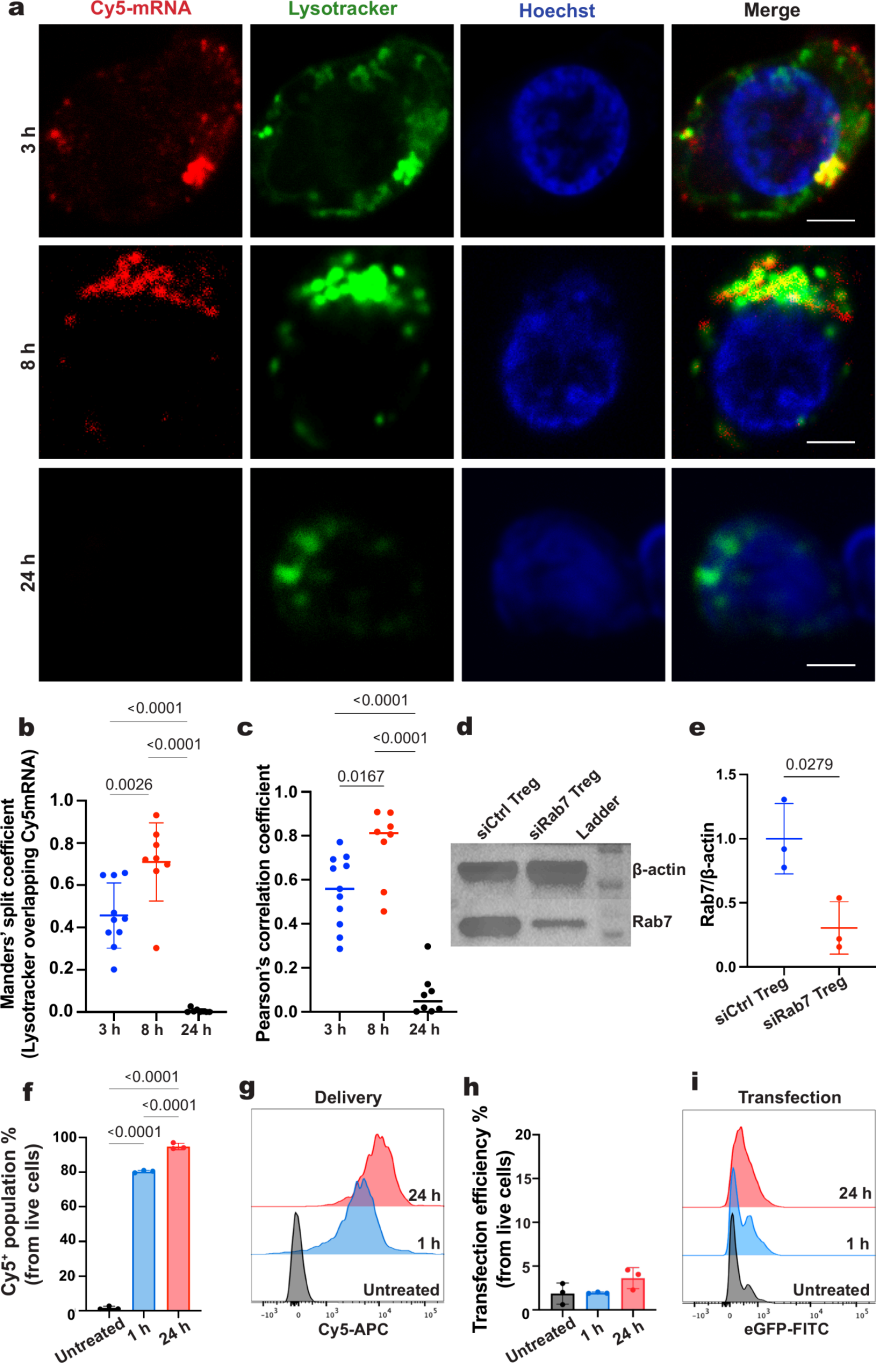
Endolysosomal trafficking of mRNA. (a) Representative confocal microscope images of Tregs at 3, 8, and 24 h post delivery showing Cy5-mRNA (red), Lysotracker-stained lysosomes (green), and Hoechst nuclear counterstain (blue). Scale bar: 2 μm. (b) Quantification of Manders’ split coefficient indicating the fraction of lysotracker overlapping Cy5-mRNA using 8–10 cells for each group from three independent biological replicates. Data presented as mean ± SD, ordinary one-way ANOVA, followed by Tukey’s multiple comparisons test. *p*-values are indicated above the bars. (c) Quantification of Pearson’s correlation coefficient using 8–10 cells for each group from three independent biological replicates. Data presented as mean ± SD, ordinary one-way ANOVA, followed by Tukey’s multiple comparisons test. *p*-values are indicated above the bars. (d) Western blot of Rab7 protein levels in Rab7 siRNA-transfected Tregs (siRab7 Tregs) compared with scrambled siRNA-transfected Tregs (siCtrl Tregs). β-actin as the loading control. (e) Densitometric Western blot quantification of Rab7 protein levels in siRab7 Tregs compared with Ctrl Tregs. Data presented as mean ± SD, *n* = 3 independent samples, unpaired *t* test with Welch’s correction. *p*-value is indicated above the bar. (f) Delivery efficiency (Cy5^+^ population) at 1 and 24 h following nanoinjection in siRab7 Tregs. Data presented as mean ± SD, *n* = 3 independent samples, ordinary one-way ANOVA, followed by Tukey’s multiple comparisons test. *p*-values are indicated above the bars. (g) Representative flow cytometry histogram of the eGFP fluorescence for groups presented in (f). (h) Transfection efficiency (Cy5^+^ eGFP^+^ population) at 1 and 24 h following nanoinjection in siRab7 Tregs. (i) Representative flow cytometry histogram of the eGFP fluorescence for groups presented in (h).

To test whether endolysosomal activity accounted for the loss of mRNA, we tracked the mRNA signal in Tregs following Rab7 silencing. Rab7 is a key GTPase regulating late endosome–lysosome maturation, and its depletion impairs trafficking to degradative and secretory pathways.^57,58^ Western blot analysis confirmed the successful knockdown of Rab7, showing a marked reduction in protein expression in siRab7-transfected Tregs (siRab7 Treg) compared with nontargeting controls (siCtrl Treg) (Figures 3d and S5). Densitometric quantification revealed a more than 2-fold decrease in Rab7 levels in siRab7 Treg relative to siCtrl Treg (Figure 3e). We then assessed the Rab7 silencing effect on Cy5-mRNA delivery. Delivery efficiency at 1 h (80% ± 1%) was comparable to wild-type (WT) Tregs (70% ± 18%), but by 24 h, siRab7 Tregs retained the delivered payload (95% ± 2%), in contrast to the marked reduction observed in WT Tregs (28% ± 11%) (Figure 3f,g). However, such endosomal retention did not translate into successful transfection (Figure 3h,i).

The loss of full-length eGFP mRNA over time, its increasing colocalization with lysosomal compartments, and mRNA retention following Rab7 silencing collectively indicate that nanoneedle-delivered mRNA in Tregs is progressively trafficked to lysosomes, where it undergoes degradation or secretion rather than cytosolic release for translation. Such endolysosomal routing constitutes a major barrier to productive nanoneedle-mediated transfection in Tregs.

### 2.4 Treg Endocytosis Pathways

To identify the uptake routes underlying nanoneedle delivery and their relationship with endolysosomal trafficking in Tregs, we investigated the roles of CLME, CavME, and macropinocytosis.

We first assessed whether nanoneedles could enhance the uptake of pathway-specific cargos in Tregs. Nanoneedles significantly increased the internalization of transferrin (Tfn), a CLME-specific cargo, as well as dextrans, which are internalized via macropinocytosis (Figure 4a–f). These results suggest that both CLME and macropinocytosis are upregulated upon nanoneedle interfacing with Tregs.

**Figure 4:**
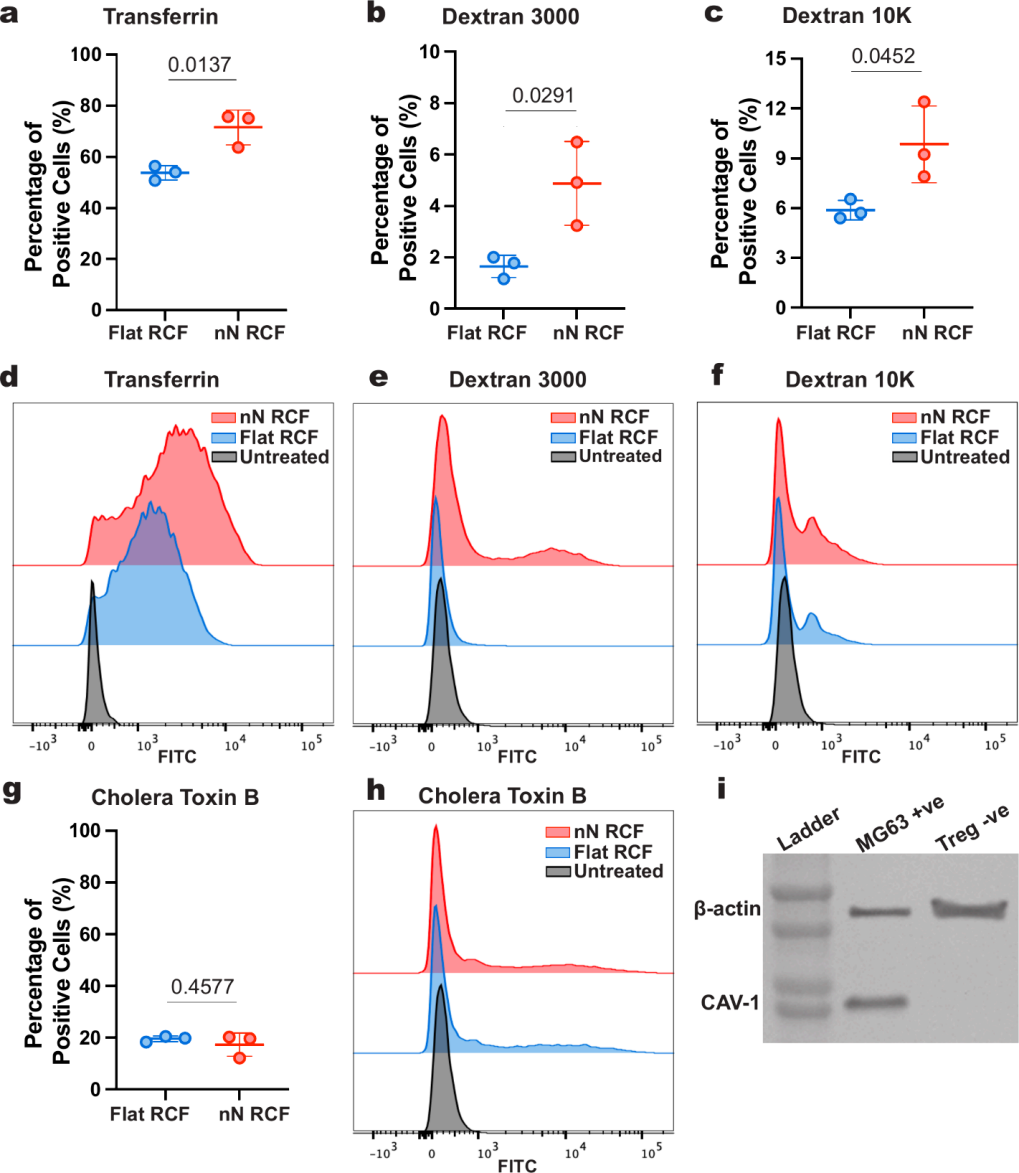
Caveolae-mediated endocytosis is inactive in Tregs. (a–c) Delivery efficiency on Flat RCF and nN RCF for the pathway-specific cargoes (a) transferrin (Tfn), (b) Dextran 3000, and (c) Dextran 10k. Data presented as mean ± SD, *n* = 3 independent samples, unpaired *t* test, *p*-value is indicated above the bars. (d–f) Representative flow cytometry histograms for the data presented in (a–c). (g) Delivery efficiency on Flat RCF and nN RCF for the caveolae-specific cargo cholera toxin B (CTxB). Data presented as mean ± SD, *n* = 3 independent samples, unpaired *t* test, *p*-value is indicated above the bars. (h) Representative flow cytometry histograms for the data in (g). (i) Western blot of Caveolin-1 (CAV-1) expression in MG63 +ve and Treg −ve, with β-actin as loading control.

In contrast, nanoneedles did not increase the uptake of cholera toxin subunit B (CTxB), a cargo internalized by CavME (Figure 4g,h). Furthermore, the uptake of CTxB in both nanoneedle-treated and control Tregs remained minimal, indicating that CavME is inactive in these cells, regardless of nanoneedle interaction.

This observation is consistent with previous reports showing that caveolins, the structural proteins essential for CavME, are virtually absent in lymphocytes.^47^ To confirm this, we performed Western blot analysis to assess the expression of CAV-1, the master regulator of CavME. As expected, CAV-1 expression was undetectable in Tregs (Treg −ve), whereas MG63 +ve, a CAV-1-expressing adherent cell line, displayed robust expression (Figures 4i and S6).

Considering the inactivity of CavME and the absence of CAV-1 expression in Tregs, as well as the respective roles of CLME in directing cargo to endolysosomal degradation^42,59^ and CavME in supporting efficient polyplexes transfection^60^ and viral transduction,^45,61^ we hypothesized that the absence of caveolae-mediated uptake contributed to the inefficient nanoneedle transfection observed in these cells.

### 2.5 Role of CAV-1 in Nanoneedle Transfection

To directly investigate the role of CAV-1 and CavME in nanoneedle transfection, we generated a CRISPR-Cas9 clonal knockout of CAV-1 in MG63 cells, a cell line known for its efficient transfection via nanoneedles (Figure S7). Western blot analysis confirmed successful CAV-1 knockout in two independent clones (MG63 −ve C1 and C2) (Figures 5a and S8).

**Figure 5:**
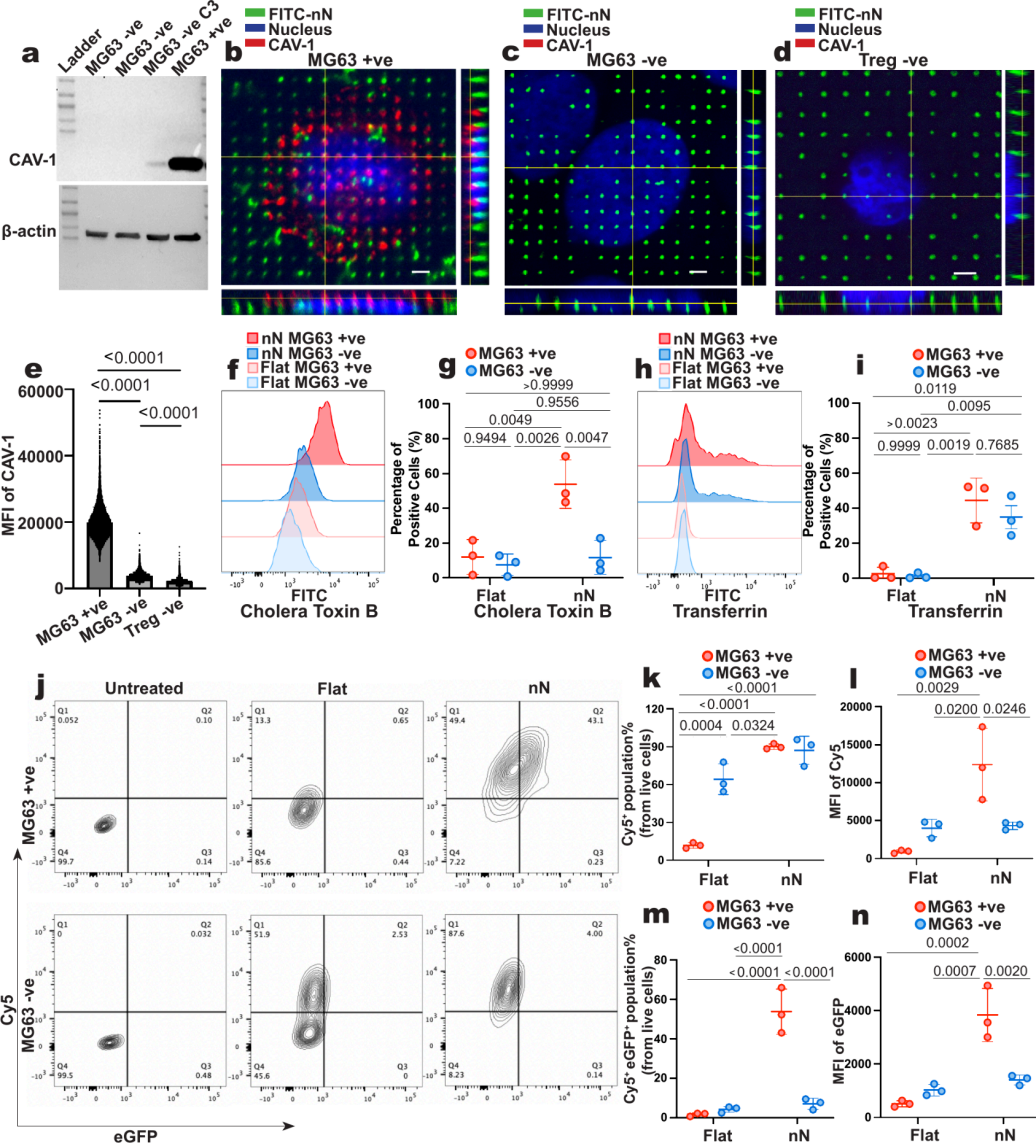
Caveolin 1 knockout abolishes nanoneedle transfection in MG63 cells. (a) Western blot of CAV-1 expression in MG63 knockout cell clones (MG63 −ve C1, C2, and C3) and MG63 (MG63 +ve). (b–d) Representative confocal fluorescence image of CAV-1 expression and localization in (b) MG63 +ve, (c) MG63 −ve, and (d) Treg −ve cells after 6 h incubation. Scale bar: 2 μm. (e) CAV-1 expression levels in MG63 +ve, MG63 −ve, and Tregs. One-way ANOVA followed by Tukey’s multiple comparisons test. *p*-values are indicated above the bars. (f) Representative flow cytometry histograms of CTxB uptake in MG63 +ve and MG63 −ve for Flat and nN. (g) Cholera toxin B (CTxB) delivery efficiency in MG63 +ve and MG63 −ve for Flat and nN. Data presented as mean ± SD, *n* = 3 independent samples, two-way ANOVA followed by Tukey’s multiple comparisons test. *p*-values are indicated above the bars. (h) Representative flow cytometry histograms of transferrin (Tfn) uptake in MG63 +ve and MG63 −ve for Flat and nN. (i) Tfn delivery efficiency in MG63 +ve and MG63 −ve for Flat and nN. Data presented as mean ± SD, *n* = 3 independent samples, two-way ANOVA followed by Tukey’s multiple comparisons test. *p*-values are indicated above the bars. (j) Representative flow cytometry contour plots of the delivery (*Y*-axis, Cy5 fluorescence) and transfection (*X*-axis, eGFP fluorescence) of Cy5-eGFP in MG63 +ve and MG63 −ve for Flat and nN. (k) Delivery efficiency (Cy5^+^ cell population: Q1 + Q2) in MG63 +ve and MG63 −ve for Flat and nN. Data presented as mean ± SD, *n* = 3 independent samples, two-way ANOVA followed by Tukey’s multiple comparisons test. *p*-values are indicated above the bars. (l) MFI values of Cy5 for groups presented in (k). Data presented as mean ± SD, *n* = 3 independent samples, two-way ANOVA followed by Tukey’s multiple comparisons test. *p*-values are indicated above the bars. (m) Transfection efficiency (Cy5^+^ eGFP^+^ cell population: Q2) in MG63 +ve and MG63 −ve for Flat and nN. Data presented as mean ± SD, *n* = 3 independent samples, two-way ANOVA, followed by Tukey’s multiple comparisons test. *p*-values are indicated above the bars. (n) MFI values of eGFP fluorescence for groups presented in (m). Data presented as mean ± SD, *n* = 3 independent samples, two-way ANOVA, followed by Tukey’s multiple comparisons test. *p*-values are indicated above the bars.

Immunofluorescence analysis revealed that in MG63 +ve cells, CAV-1 clustered around nanoneedles during interfacing, indicating active CavME engagement (Figure 5b). As expected, CAV-1 was absent in both MG63 −ve cells and Treg −ve cells, as confirmed by fluorescence imaging (Figure 5c–e). Morphologically, MG63 −ve cells exhibited reduced adhesion and slower spreading within the first 2 h postseeding, likely due to impaired focal adhesion dynamics. However, by 24 h, their morphology was comparable to that of MG63 +ve cells (Figure S9).

We next assessed whether the loss of CAV-1 affected the endocytic activity during nanoinjection. In MG63 +ve cells, nanoneedles significantly enhanced the uptake of CTxB (54% ± 14%) as well as Tfn (44% ± 13%) compared to the planar controls. In contrast, MG63 −ve cells showed a dramatic reduction in CTxB uptake (12% ± 10%), confirming the disruption of CavME. Importantly, Tfn uptake remained comparable between MG63 +ve and MG63 −ve cells, indicating that CLME remained unaffected by CAV-1 knockout (Figure 5f–i). These findings indicate that CAV-1 selectively regulates CavME activation without impacting clathrin-mediated pathways.

We then examined whether CAV-1 loss impacted nanoneedle delivery and transfection efficiency in MG63 cells (Figure 5j). The delivery efficiency of Cy5-eGFP was comparable in MG63 +ve and MG63 −ve cells (90% ± 2% and 87% ± 11% respectively), significantly higher than control (Figure 5k). However, the Cy5 MFI value was significantly lower in MG63 −ve cells (4325 ± 497) than in MG63 +ve cells (12373 ± 4804), indicating that CavME contributes substantially (around 65%) to overall mRNA delivery (Figure 5l).

In MG63 +ve cells, nanoneedles achieved a transfection efficiency of 54% ± 12% (MFI: 3834 ± 993), compared to 2% ± 1% (MFI: 513 ± 114) for the flat control. However, in MG63 −ve cells, nanoneedle transfection efficiency dropped dramatically to 7% ± 3% (MFI: 1408 ± 185), despite the preserved delivery efficiency (Figure 5m,n).

Although MG63 +ve and MG63 −ve cells exhibited comparable mRNA delivery efficiency on nanoneedles, the transfection efficiency was markedly reduced in MG63 −ve cells. Furthermore, the lower Cy5 MFI value in MG63 −ve cells highlights that CavME contributes to the total amount of mRNA delivered. This indicates that alternative uptake mechanisms can sustain delivery in the absence of CAV-1, but while not required for delivery, CAV-1 is indispensable for intracellular trafficking and processing leading to gene expression.

To confirm that the loss of nanoneedle-mediated transfection observed following CAV-1 knockout reflects disruption of the CavME pathway, rather than pleiotropic, CAV-1–specific effects on cell physiology, we silenced polymerase I and the transcript release factor (PTRF/Cavin-1), another essential component of caveolae. PTRF partners with CAV-1 to stabilize the caveolar coat; its acute depletion disassembles caveolae and selectively impairs CavME while leaving other caveolin-dependent functions largely intact. Western blot confirmed the reduced PTRF expression level (Figures S10a,b and S11). As expected, delivery efficiency remained high (99% ± 1%) in siPTRF MG63, whereas transfection efficiency significantly decreased (3% ± 2%) to levels comparable with MG63 −ve due to impaired caveolar trafficking (Figure S10c–e).

To further assess the involvement of endolysosomal trafficking in the intracellular fate of delivered mRNA, we silenced Rab7 in MG63 +ve cells to disrupt late lysosomal maturation (Figures S12a,b and S13). Following nanoinjection, the delivery efficiency remained 98% ± 1%, and the transection efficiency was 35% ± 4% at 24 h, comparable to wild type MG63 (Figure S12c–e). This preserved transfection efficiency despite impaired lysosomal maturation demonstrates that gene expression proceeds independently of endolysosomal processing.

These data show that in MG63 cells, efficient transfection correlates with active CavME, while CavME disruption by CAV-1 knockout or PTRF silencing significantly reduces the transfection efficiency. However, disruption of lysosomal maturation by Rab7 knockout does not impact the transfection efficiency.

Taken together, these results demonstrate that in MG63 cells, nanoneedle-mediated delivery relies on CavME to achieve efficient transfection, whereas the CLME pathway is dispensable for gene expression and mainly associated with degradative trafficking.

### 2.6 CAV-1 Governs Nanoneedle Transfection in Tregs

To assess the role of CAV-1 in nanoneedle transfection of Tregs, we generated CAV-1 knock-in Tregs (Treg-KI) by introducing CAV-1-mCherry via nucleofection,^56^ achieving a cell viability of 74% ± 4% (Figure 6a,b) and a transfection efficiency of 24% ± 7% (Figure 6c,d). In the unsorted Treg-KI cell pool, nanoneedle interfacing significantly increased CTxB uptake to 65% ± 5%, compared to 20% ± 2% in Tregs (Treg −ve), indicating activation of CavME (Figure 6e,f).

**Figure 6:**
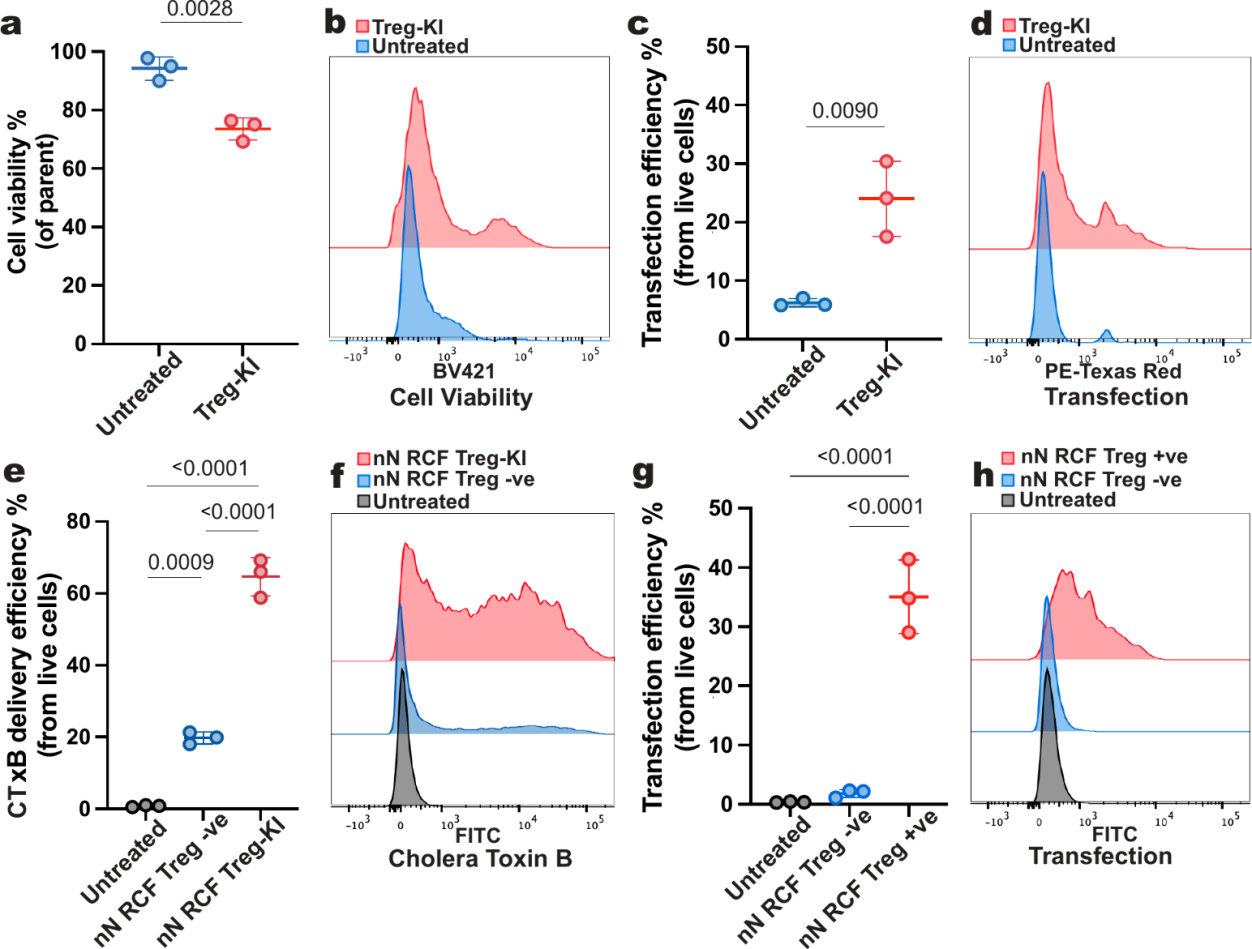
Caveolin 1 knock in induces nanoneedle transfection in Tregs. (a) Cell viability after nucleofection of CAV-1-mCherry into Tregs (Treg-KI) compared to untreated control. Data presented as mean ± SD, *n* = 3 independent samples, unpaired *t* test, *p*-value is indicated above the bar. (b) Representative flow cytometry histogram for groups presented in (a). (c) Fraction of Treg-KI expressing mCherry compared to the untreated control. Data presented as mean ± SD, *n* = 3 independent samples, unpaired *t* test, *p*-value is indicated above the bars. (d) Representative flow cytometry histogram for groups presented in (c). (e) Uptake of caveolae-specific cargo cholera toxin B (CTxB) in nN RCF Treg-KI compared to the nN RCF Treg control (nN RCF Treg −ve). Data presented as mean ± SD, *n* = 3 independent samples, ordinary one-way ANOVA, followed by Tukey’s multiple comparisons test, *p*-values are indicated above the bars. (f) Representative flow cytometry histogram for groups presented in (e). (g) Nanoneedle transfection efficiency for purified Treg-KI (nN RCF Treg +ve) and nN RCF Treg −ve. Data presented as mean ± SD, *n* = 3 independent samples, unpaired *t* test, and *p*-values are indicated above the bars. (h) Representative flow cytometry histogram for groups presented in (g).

We sorted the Treg-KI for their mCherry expression to obtain a pure population of CAV-1-expressing Tregs (Treg +ve) (Figure S14). Nanoneedle transfection of Treg +ve cells reached 35% ± 6% efficiency, a substantial increase compared to 2% ± 1% for Treg +ve (Figure 6g,h). These results demonstrate that CAV-1 expression governs nanoneedle transfection in primary human Tregs by activating CavME.

To confirm the generality of this behavior, we extended our analysis to MCF-7 cells, an adherent cancer cell line that exhibits markedly lower CAV-1 expression and fewer plasma membrane caveolae (Figures S15a and S16).^62^ Consistent with the results in Tregs and MG63 −ve cells, nanoinjection of Cy5-eGFP in MCF-7 cells displayed a high delivery efficiency (Figure S15b,c) but no detectable eGFP expression (Figure S15d,e). Together, these findings reinforce the conclusion that although caveolae are not required for nanoneedle-mediated cargo delivery, they are essential for the downstream processing steps leading to functional transfection.

## 3 Conclusions

Nanoneedles have already demonstrated efficient gene delivery in a variety of primary and hard-to-transfect cell types.^1–3,8,11,12,26,35,63–65^ By identifying CAV-1-dependent CavME as essential for transfection, this work establishes a mechanistic basis to rationally enhance nanoneedle platforms and expand their applicability in gene therapy. Our findings show that while nanoneedles can efficiently deliver genetic material into cells, transfection, defined by transgene expression, occurs only when CavME is active. In Tregs, which lack endogenous CAV-1 and thus CavME activity, the delivered mRNA is routed to lysosomes and degrades within 24 h, preventing gene expression. Attempts to enhance endosomal escape using PEI functionalization failed to overcome this barrier.

In contrast, inducing CAV-1 expression in Tregs to establish CavME markedly increased transfection efficiency, whereas CAV-1 knockout in MG63 cells, normally highly transfectable, abolished transgene expression despite preserved delivery. PTRF silencing produced a similar outcome, confirming that the effect arises specifically from caveolae disruption rather than from broader CAV-1 functions. Impairing endosomal maturation by Rab7 silencing enhanced mRNA retention at 24h (in Tregs) but did not impact transfection efficiency, indicating that the endolysosomal route played no significant role in transfection.

Overall, our findings support a model in which nanoneedle engagement of CLME directs the delivered payload to endolysosomal degradation, whereas engagement of CavME enables productive transfection. This aligns with the established roles of these pathways: CavME promotes nondegradative trafficking that allows internalized cargo to bypass lysosomes and access compartments supporting gene expression,^66^ while CLME typically routes cargo to lysosomal compartments for degradation.^67^ Previous studies have likewise shown that polyplexes internalized via CavME achieve efficient transfection, unlike those entering through CLME.^60,42^

Future studies should focus on engineering nanoneedle interfaces or modulating endocytic signaling to activate CavME-like pathways in caveolae-deficient cells, broadening the therapeutic scope of nanoneedle-based gene delivery.

## 4 Experimental Section

### 4.1 Fabrication of the Porous Silicon Nanoneedles

Porous silicon nanoneedles were manufactured based on our established protocol.^2,64^ To finalize the nanoneedle structure, a back-end reactive ion etching step was conducted in SF_6_ plasma at 100 mTorr, 300 W, and 20 sccm for 220 and 240 s, yielding nanoneedles with heights of 3 and 2 μm, respectively. For the fabrication of 6 μm nanoneedles, the MACE duration was increased to 20 min instead of 7 min 30 s, followed by 180 s RIE at 100 mTorr, 300 W, and 20 sccm. The substrates were diced into 8 × 8 mm chips using a DISCO DAD3230 dicing saw (Japan).

### 4.2 Human Primary Tregs and Teffs Cell Isolation and Culture

Peripheral blood from anonymized healthy donors was obtained from NHS Blood and Transplantation (Tooting, London, UK) with informed consent and ethical approval granted by the Institutional Review Board of Guy’s Hospital (reference 09/H0707/86).

A GMP-compliant method as published before was employed to isolate CD4^+^CD25^+^ Tregs and CD4^+^CD25^–^ Teffs from leukocyte-enriched blood cones. After isolation, Tregs were counted as Day 0 Tregs and activated with anti-CD3/CD28 Dynabeads at a 1:1 bead-to-cell ratio (ThermoFisher, UK). The cell culture in X-VIVO medium was supplemented with 1000 IU/mL recombinant human IL-2 (RD, Minnesota, USA) and 100 nM rapamycin (LC-Laboratories, MA, USA). Cell culture medium was half-changed every 48 h.

### 4.3 Treg Suppression Assay

The suppressive function of Tregs after nN RCF treatment was measured in vitro by assessing the efficacy with which these cells inhibited the proliferation of CD4^+^CD25^–^ Teffs. Teffs were labeled with 2 μM CellTrace Violet (ThermoFisher Scientific, USA) according to manufacturer’s instructions. Then, antihuman CD3/CD28 Dynabeads were added to the Teffs at a 1:40 bead-to-cell ratio. Day3 Tregs after nN RCF treatment were harvested, and Dynabeads were removed using a magnet. Teffs were cocultured with a varying number of Tregs to achieve the desired Teffs: Tregs ratio in 96-well U-bottom plates in a final volume of 200 μL for 5 days, after which Teffs proliferation was measured by flow cytometry LSRFortessa II (BD).

### 4.4 Cell Metabolism ATP Assay

The ATP determination kit was used to confirm that nanoneedles minimize perturbation to the metabolic states of cells. Cells after nanoinjection were collected and spun down in well plates. Then the cells were incubated in ATP-2D working solution (100 μL of phenol-free medium and 100 μL of the ATP-2D for each sample) on a shaker (250 rpm for 2 min). Plates were left on the bench for 10 min for the signal to equilibrate. Transfer cells to a 96-well black plate to read the luminescence via Clariostar.

### 4.5 Caspase-3/7 Apoptosis Assay

Cell apoptosis was assessed using the Caspase-3/7 assay kit green/red (ThermoFisher, USA) following the manufacturer’s instructions. Tregs were incubated with caspase-3/7 reagent for 1 h in the dark, washed once with phosphate-buffered saline (PBS), and resuspended in 200 μL of PBS for flow cytometry analysis.

### 4.6 Flow Cytometric Phenotype Analysis

Day3 Tregs after nanoinjection were collected for phenotyping analysis. For extracellular marker staining, cells were incubated with fluorescently conjugated antibodies (CD4, SK3 clone, BioLegend; CD25, BC96 clone, BioLegend; CD127, A019D5 clone, BioLegend) and the LIVE/DEAD cell stain reagent (Invitrogen, USA) at 4 °C for 30 min. Then cells were fixed and permeabilized using the Foxp3/Transcription Factor Fixation/Permeabilization Kit (Invitrogen, USA) for 1 h at 4 °C. After fixation, samples were washed with a 1:10 dilution of permeabilization buffer in water for 10 min and resuspended in 100 μL of permeabilization buffer containing intracellular antibodies (Foxp3, 206D clone, BioLegend; *K*_i_-67, *K*_i_-67 clone, BioLegend; CTLA-4, BNI3 clone, BioLegend; CD69, FN50 clone, BioLegend) and incubated for 1 h at 4 °C. Compensation controls were prepared using CompBeads (BD, USA).^68^

### 4.7 Scanning Electron Microscopy Imaging

Cells cultured on nanoneedle substrates were washed three times with PBS for 5 min per wash before being fixed in 4% paraformaldehyde (PFA) at 4 °C overnight. After fixation, the substrates were rinsed three times with chilled Milli-Q water at room temperature for 5 min each. A stepwise dehydration process was performed using ethanol solutions of increasing concentrations: 30, 50, 70, 90, and 96% (each for 10 min), followed by two washes in 100% ethanol for 10 min each at room temperature. The samples were subsequently mounted onto SEM stubs and coated with a 5 nm gold layer via sputtering to improve the conductivity.

### 4.8 FITC Labeling Nanoneedle Array

Nanoneedle chips were treated with oxygen plasma (100 W, ZEPTO-W6, Diener Electronic) to activate their surface. Following this, the chips were incubated in a 2% APTES solution (in absolute ethanol) for 3 h at room temperature. The modified chips were thoroughly washed three times with absolute ethanol, and then the nanoneedle chips were incubated in a 1 mM FITC solution (prepared by dissolving 0.4 mg of FITC in 1.0 mL of PBS) for 2 h at room temperature. The chips were washed three times with deionized water, followed by air-drying.

### 4.9 Nanoinjection of Tregs

Nanoneedle chips (8 × 8 mm) were treated with oxygen plasma (100 W, ZEPTO-W6, Diener Electronic) and then incubated in 0.1 mg/mL poly-l-lysine (PLL) (Sigma) for 1 h. After the chips were rinsed twice in distilled water, Cy5-eGFP was loaded on the chips and incubated for 30 min. The distribution of Cy5 was visualized under a Leica fluorescent microscope. Nanoneedle chips were placed in the chamber slides. Tregs were activated for 3 days and centrifuged on top of the nanoneedle chips (300,000 cells per well) by using swinging bucket centrifugation at 37 °C, 600 RCF for 30 min. After culturing for 48 h, Tregs were collected from nanoneedle chips by gently pipetting and stained with the LIVE/DEAD reagent. Samples were analyzed via flow cytometry to assess the viability, delivery efficiency, and transfection efficiency.

### 4.10 Nanoneedle Transferrin-PEI Transfection

The transfection mixture was prepared using the transferrin-PEI (Invitrogen, USA) kit according to the manufacturer’s instructions.^69,70^ Then, the transfection mixture was loaded onto nanoneedle chips. Day3 Tregs were centrifuged on the nanoneedles at 600 RCF for 30 min. Cells were collected after incubation for 48 h for analysis.

### 4.11 Nanoneedle Silane-PEG NHS PEI Transfection

Nanoneedle chips were oxygen plasma-treated, and then Silane-PEG-NHS (NANOCS. PG2-NSSL-5k) was conjugated onto the nanoneedle surface. Branched PEI (Sigma-Aldrich,9002-98-6) was reconstituted with 10 wt/v% in deionized water and spun coated on the silane-PEG-NHS treated surfaces at 1000 rpm for 30s. After that, samples were incubated for 30 min at room temperature, followed by thermal annealing at 70 °C on a hot plate for 5 min. Samples were rigorously washed with ethanol and deionized water and air-dried.^14^

### 4.12 Reverse Transcription and Quantitative Polymerase Chain Reaction

Nanoneedle-transfected Tregs were collected at 1, 16, and 48 h after nanoinjection. Total RNA was extracted from cells using an RNeasy mini kit (Qiagen). Reverse transcription to cDNA was performed using ProtoScript II First Strand cDNA Synthesis Kit according to the manufacturer’s protocol. Quantitative polymerase chain reaction (PCR) was performed with a LightCycler96 Instrument (Roche, USA). The primer sequences for eGFP were: Forward (5′-3′): TTCAAGGACGACGGCAACTAC; Reverse (5′-3′): GTGCCCCAGGATGTTGCCGT. The relative expression of the target gene was normalized to housekeeping gene Hypoxanthine-Guanine Phosphoribosyltransferase (HPRT) using the 2^–ΔΔCT^ method. The results at 16 and 48 h were further normalized to the 1 h value.

### 4.13 Lysotracker Staining

For live-cell lysosomal labeling, Tregs were incubated with LysoTracker Red DND-99 dye (100 nM, Thermo Fisher Scientific) diluted in prewarmed cell culture medium at 37 °C for 2 h, protected from light. Hoechst 33342 (1 μg/mL, Thermo Fisher Scientific) was added during the last 15 min of staining for nuclear counterstaining. Samples were washed once in PBS before confocal imaging. Colocalization analysis between LysoTracker and Cy5 signals was carried out using Fiji/ImageJ. Manders’ coefficient and Pearson’s correlation coefficient were calculated using 8–10 cells per group from three independent biological replicates. Colocalization was quantified using the JaCoP plugin,^71^ reporting Manders’ split coefficient (Lysotracker overlapping Cy5-mRNA) and Pearson’s correlation coefficient. Thresholds for each channel were determined by JaCoP using the same algorithm and settings for all of the images.

### 4.14 Gene Silencing

MG63 cells were plated in 12-well plates 24 h before siRNA transfection. Cells were transfected with control siRNA (siCtrl), Rab7 siRNA (siRab7), and PTRF siRNA (siPTRF) using Lipofectamine RNAimax (Thermo Fisher Scientific) when the cell confluency was 80% according to the manufacturer’s protocol. Tregs were transfected with siCtrl and siRab7 using the Lonza nucleofector program EH-115. Cells were recovered and incubated for 24 h before further experiments.

### 4.15 Generation of CAV-1 Knockout Single Cell Clones in MG63

We utilized the CRISPR-Cas9 system to knock out the CAV-1 gene in MG63 +ve cells. eGFP-tagged Cas9 nuclease mRNA was purchased from Horizon for cotransfection with synthetic guide RNA (sgRNA). The CAV-1 sgRNA target sequence (AUGUUGCCCUGUUCCCGGAU) was ordered from Synthego. Approximately 80,000 MG63 +ve cells were plated 24 h prior to transfection in a 12-well plate. The transfection solution, containing 100 μL of opti-MEM (Thermo Scientific), 1 μg of sgRNA, 1 μg of Cas9 mRNA, 1 μL of mRNA boost reagent, and 1 μL of TransIT-mRNA reagent (Mirus), was then added to each well. Cells were incubated for 48 h before being harvested and prepared for FACS sorting. Single-cell clones were isolated and expanded. Gene function was subsequently tested by Western blotting.

### 4.16 Sanger Sequencing

To determine the editing efficiency in the single cell-derived colonies, 50% of cells were collected and spun down; the supernatant was discarded. Genomic DNA was extracted using the QIAamp DNA mini kit according to the manufacturer’s protocol and measured using a NanoDrop spectrophotometer. The CAV-1 forward and reverse primer sequences are shown below. The PCR product was purified from excess dNTPs and unincorporated primers using Illustra ExoProStar (Cytiva) according to the manufacturer’s protocol. The thermal cycler program was set as follows: 37 °C for 15 min, 80 °C for 15 min, and infinity at 10 °C. Sanger sequencing was carried out by an external company (SourceBioscience), and the sequencing results were analyzed using the Inference of CRISPR Edits (ICE) tool.Primer namePrimer
sequence (5′-3′)CAV-1_ForwardGTGTGGTGTCCTCTGCGAGACAV-1_ReverseTGTGTGTGTGTGTGTGTGCGCAV-1_SequencingAAGAAGGATGCACGGGCTAACTG


### 4.17 Western Blot

Cells were rinsed once in PBS and lysed on ice with 100 μL of RIPA lysis buffer (Sigma) with 1% Proteinase Inhibitor Cocktail (Calbiochem). The lysate was stored at −80 °C overnight and subsequently centrifuged at 8000 *g*, 4 °C for 10 min to remove cell debris. The total protein content was measured using a Pierce BCA Protein Assay Kit (Thermo Scientific) and boiled at 95 °C for 10 min with 4 × Sample buffer (Bio-Rad) containing 10% β-mercaptoethanol at a 3:1 ratio. Then, 20 μg of protein from each sample was separated using 10% sodium dodecyl sulfate polyacrylamide gel electrophoresis (SDS-PAGE). The turbo transfer system (Bio-Rad) was used to transfer the proteins from the gel to a PVDF membrane at 25 V for 10 min. The membranes were blocked with 5% milk PBS-T for 1 h at room temperature and then incubated with primary antibodies: anti-CAV-1 (Cell signaling) (1:1000 dilution), anti-PTRF antibody (Thermo Fisher Scientific, 1:2000 dilution), anti-Rab7 antibody (Thermo Fisher Scientific, 1:2000 dilution), anti-β-actin (Santa-Cruz Biotechnologies) (1:2000), and anti-HSP (heat shock protein) 90 (Thermo Fisher Scientific, 1:2000 dilution). The respective secondary antibodies were incubated for 1 h at room temperature [goat antirabbit horseradish-peroxidase (HRP) (Dako, 1:2000) and donkey antimouse HRP (Dako, 1:2000)]. The membranes were developed for 2 min in the dark using the SuperSignal West Femto Maximum Sensitivity Substrate (Thermo Scientific) kit for CAV-1 and the ECL Prime Western Blotting Detection Reagent (Cytiva) for β-actin. Finally, the membrane was imaged on an iBright FL1500 imaging system.

### 4.18 CAV-1 Quantification

Cellpose 3.0.8 was utilized for cellular segmentation. Scikit-image 0.25.0, pandas 2.2.3, opencv 4.10.0, and numpy 2.0.0 were used to quantify the fluorescent intensity of CAV-1 and relevant morphometry using the masks generated by Cellpose 3.0.8. The distributions of cell area were plotted, and cells were filtered within the 5th and 95th percentiles of these Gaussian distributions to account for outliers. Data sets were generated for CAV-1 intensity for these filtered cells and plotted using Graphpad Prism 10.

### 4.19 Nanoneedle-Mediated Biomolecule Delivery

Fluorescently labeled Transferrin (25 μg/mL), Cholera Toxin B (5 μg/mL), and Dextrans (50 μg/mL; Dextran 3000 and Dextran 10K) were reconstituted in a buffer containing 0.25 M glycine and 400 mM KCl, with pH adjusted to 5.0. Each solution (10 μL) was loaded onto nanoneedle chips and incubated for 45 min at room temperature. Nanoneedle chips were then washed once and transferred to chamber slides. To assess the uptake in MG63 +ve and MG63 −ve, 50 μL cell suspension containing 20,000 cells was seeded onto each nanoneedle chip. After 2 h, once cells had attached to the substrates, the cell culture media was gently topped up to 300 μL per well. To assess the uptake in Tregs, 300,000 Tregs in 300 μL of X-VIVO medium were added on top of the nanoneedle chips in each well, followed by centrifugation at 37 °C, 600 RCF, 30 min. Cell samples were collected after 6 h to assess delivery efficiency by flow cytometry. Data were analyzed using FlowJo, and an equal population gating strategy was utilized for comparison across experimental groups.

### 4.20 Nanoinjection of MG63 Cells in Vitro

MG63 +ve and MG63 −ve cells (20,000 cells per well) were seeded separately onto chamber slides 24 h before transfection to reach 80% cell confluency. The nanoneedle chips were applied facing down over the cell monolayer, where medium was removed and spun at 37 °C, 350 RCF for 30 min in a swinging bucket rotor. 300 μL of Opti-MEM was added to the well, and chips were removed after 1 h of incubation. Cells were collected by trypsinization after 48 h and stained with LIVE/DEAD reagent to check viability while measuring Cy5 delivery and eGFP expression using flow cytometry.

### 4.21 Confocal Image Showing Cy5^+^ Treg on Nanoneedle Array

The Tregs membrane was stained with WGA-647 (ThermoFisher) at a 1:1000 dilution. Subsequently, Tregs were centrifuged on top of the nanoneedle surface that was loaded with Cy5-eGFP (0.25 μL/chip). After being cultured in an incubator for 24 h, samples were fixed using 4% paraformaldehyde (PFA) for 10 min, followed by washing with PBS three times. Nanoneedle chips were flipped down and hard-mounted onto coverslips using Fluoreshield media containing DAPI (Abcam). The samples were imaged by a Z-stack confocal microscope (Zeiss LSM 980) equipped with a 63× 1.2 NA oil objective. Image analysis was performed by using Fiji software. Orthogonal projections were generated for visualization.

### 4.22 Nucleofection

Day3 Tregs were collected and washed once in PBS and then resuspended in a P3 Primary Cell 4D-Nucleofector X Kit (V4XP-3032, Lonza, Swiss). To transfect Tregs with CAV-1-mCherry, cells were transfected into the nucleofection cuvette and electroporated using the 4D-Nucleofector Core Unit (AAF-1002B, Lonza, Switzerland) and X Unit (AAF-1002X, Lonza, Switzerland) with pulse code EH-115. Treg-KI were transferred from a cuvette to well-plates and cultured for 48 h before cell sorting.

### 4.23 Cell Sorting

Treg-KI cells were collected 48 h postnucleofection and washed once in MACS buffer. Then, cells were stained with LIVE/DEAD violet reagent to determine the viability of the cells. Cells were acquired and sorted using FACSArial II (BD) and FACSDiva 9.4 software (BD). Debris, doublets, and dead cells were excluded.^55^ The mCherry +ve cell population representing CAV-1-expressing Tregs was collected in X-VIVO medium.

### 4.24 Statistical Analysis

All data were presented as mean ± standard deviation (SD) and were analyzed using GraphPad Prism software. Individual data points represent independent measurements. The error bars represent the SD of the experimental groups. The statistical methods and significance values (*p*-values) obtained were reported in the relative figure caption for each graph.

## Supplementary Material



## Notes

Flow cytometry gating strategies; nanoneedle optimization experiments; Western blot analyses; siRNA-mediated gene silencing validation; Sanger sequencing validation; quantitative PCR; and additional transfection efficiency results (PDF)
