# Supplementary material for: Caveolar Endocytosis Governs Nanoneedle Transfection
Source: ACS Nano. 2026 Feb 6;20(6):4663–76. doi: 10.1021/acsnano.5c11011 (PMC12918721; doi:10.1021/acsnano.5c11011)
Supplement: Supplementary file 1 [file nn5c11011_si_001.pdf]

Caveolar Endocytosis Governs Nanoneedle Transfection

Ningjia Sun[1,2], Cong Wang[1,3,4], Yikai Wang[1,2], William Edwards[1], Marija Dimitrievska[1,5], Yike Li[1,6,7], Nemanja Vasovic[8], Samuel McLennan[1,3], Hongting Zhu[1], Ermei Mäkilä[9], Jarno Salonen[9], Jiefei Shen[6,7], Qi Peng[2], Cristiano Scottà[2,10], Giovanna Lombardi[2], Ciro Chiappini[1,3*]

[1] Centre for Craniofacial and Regenerative Biology, King's College London, London SE1 9RT, U.K.

[2] Peter Gorer Department of Immunobiology, School of Immunology & Microbial Sciences, Faculty of Life Sciences & Medicine, King's College London, London SE1 7EH, U.K.

[3] London Centre for Nanotechnology, King's College London, London WC2R 2LS, U.K.

[4] Wenzhou Eye Valley Innovation Centre, Eye Hospital, Wenzhou Medical University, Wenzhou 325024, China

[5] St John's Institute of Dermatology, School of Basic & Medical Biosciences, King's College London, London SE1 9RT, U.K.

[6] State Key Laboratory of Oral Diseases & National Centre for Stomatology & National Clinical Research Centre for Oral Diseases, West China Hospital of Stomatology, Sichuan University, Chengdu 610041, China

[7] Department of Prosthodontics, West China Hospital of Stomatology, Sichuan University, Chengdu 610041, China

[8] Centre for Gene Therapy & Regenerative Medicine, King's College London, London SE1 9RT, U.K.

[9] Department of Physics and Astronomy, University of Turku, Turku 20014, Finland

[10] Department of Life Sciences, Centre for Inflammation Research and Translational Medicine, Brunel University London, London UB8 3PH, U.K.

*Correspondence to: ciro.chiappini@kcl.ac.uk

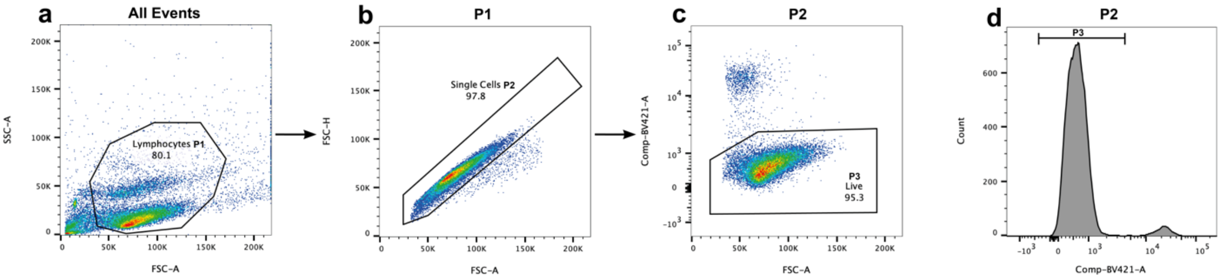

Figure S1. Representative gating strategy for cell viability analysis in Flowjo. (a) *Gating of population 1 (P1) lymphocytes from all events.* ***(b)*** *Gating of P2 single cell population from P1.* ***(c)*** *Gating of P3 viable cell population (BV421 -ve) from P2.* ***(d)*** *The histogram of BV421 channel. The gating (horizontal line) represents viable cell population.*

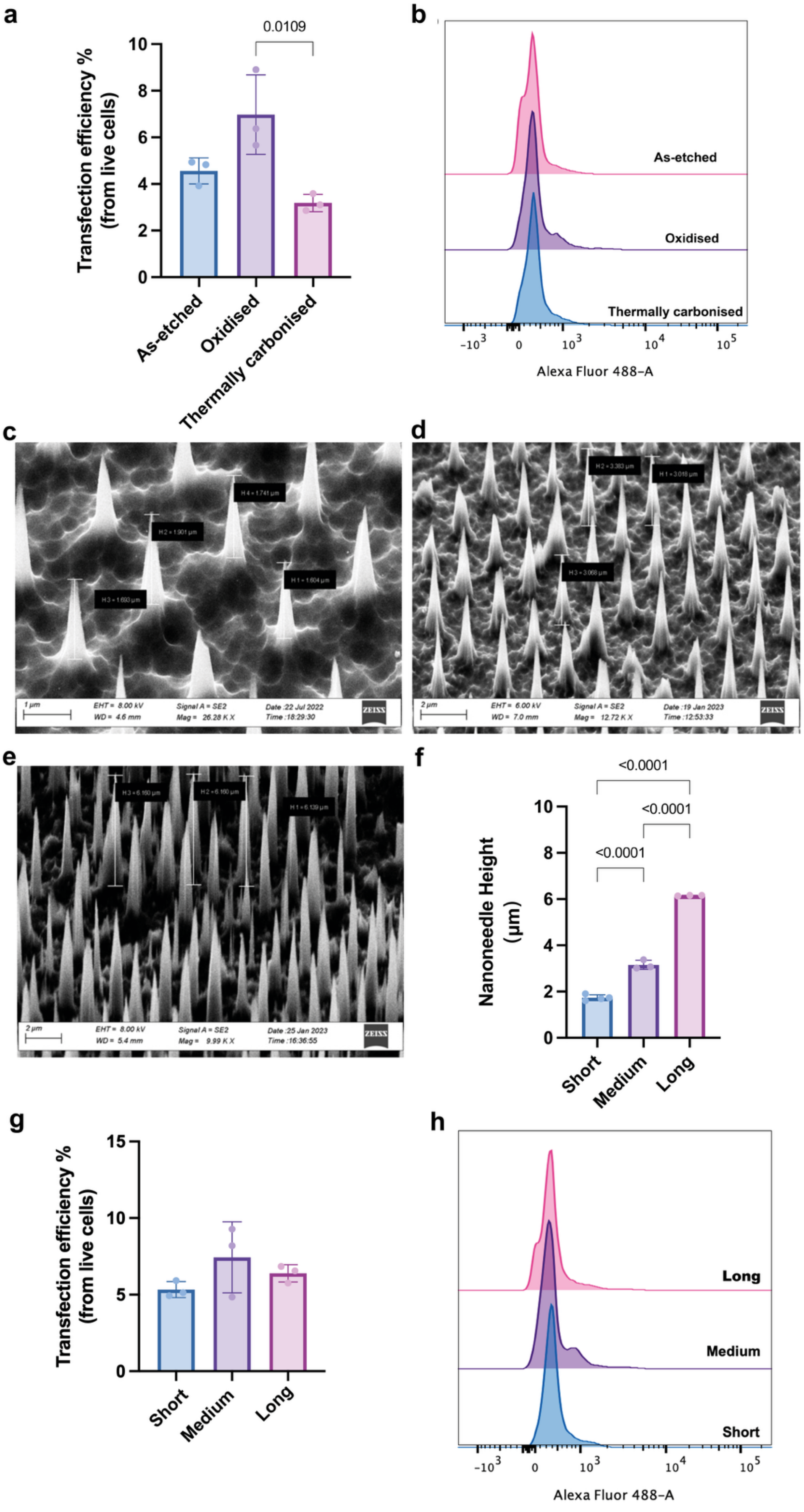
a
b
c
d
e
f
g
h
Transfection efficiency % (from live cells)
0.0109
10
8
6
4
2
0
As-etched
Oxidised
Thermally carbonised
Alexa Fluor 488-A
-10^3
0
10^3
10^4
10^5
Nanoneedle Height (µm)
<0.0001
<0.0001
<0.0001
Short
Medium
Long
15
10
5
0

Figure S2. Nanoneedle optimisation for nanoinjection. (a) *Transfection efficiency of Tregs nanoinjected using as-etched, oxidised and thermally carbonised nanoneedles. Data presented as mean ± SD, n = 3 independent samples, ordinary one-way ANOVA followed by Tukey's multiple comparisons test. p-values are indicated above the bars.* ***(b)*** *Representative flow cytometry histogram of the eGFP fluorescence for the data presented in (a).* ***(c-e)*** *SEM image of nanoneedles with* ***(c)*** *2 µm height (short),* ***(d)*** *3 µm height (medium)* ***(e)*** *6 µm height (long).* ***(f)*** *Quantification of nanoneedle height from SEM images. Data presented as mean ± SD, n = 3 independent samples, ordinary one-way ANOVA followed by Tukey's multiple comparisons test. p-values are indicated above the bars.* ***(g)*** *Quantification of transfection efficiency in Tregs using nanoneedles of varying heights. Data presented as mean ± SD, n = 3 independent samples, ordinary one-way ANOVA followed by Tukey's multiple comparisons test.* ***(h)*** *Representative flow cytometry histogram of the eGFP fluorescence for the data presented in (g).*

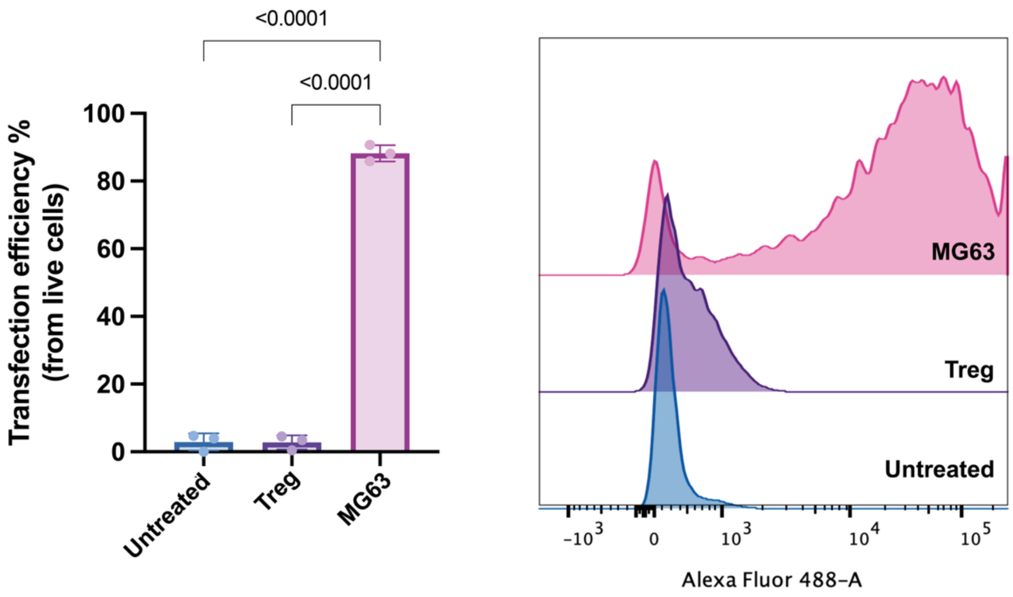

Figure S3. Lipofection of MG63 and Tregs. (a) *Quantification of transfection efficiency from flow cytometry analysis. Data presented as mean ± SD, n = 3 independent samples, ordinary one-way ANOVA followed by Tukey's multiple comparisons test. p-values are indicated above the bars.* ***(b)*** *Representative flow cytometry histogram of the eGFP fluorescence for groups presented in (a).*

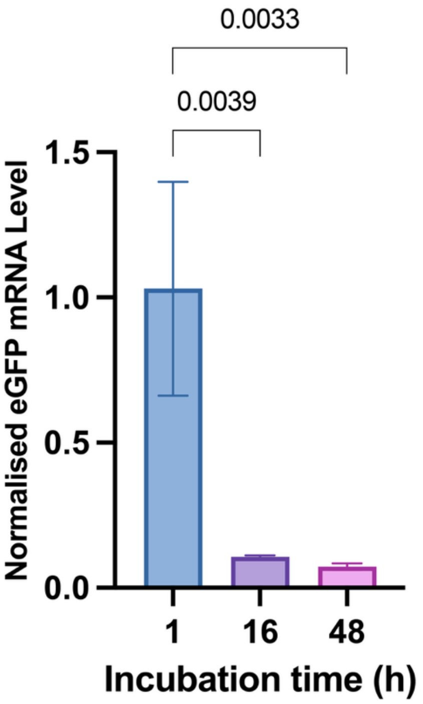

Figure S4. Intact mRNA quantification. *Relative amounts of intact eGFP mRNA at 1 h, 16 h and 48 h post nanoinjection. Data presented as mean ± SD, ordinary one-way ANOVA followed by Tukey's multiple comparisons test. p-values are indicated above the bars.*

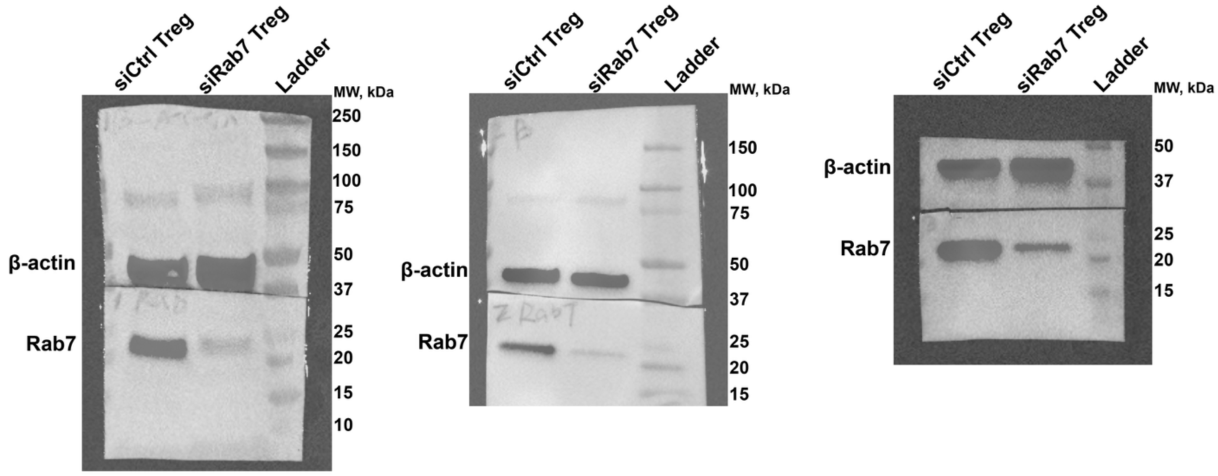

Figure S5. Western blot analysis from three independent biological replicates used for quantification shown in Figure 3 d. *Chemiluminescence detection of β-actin and Rab7 under identical exposure conditions, with the complete membrane displayed and a demarcation line indicating the separation between β-actin and Rab7 regions. Lane order on each membrane (left to right): siCtrl Treg, siRab7 Treg, and Ladder.*

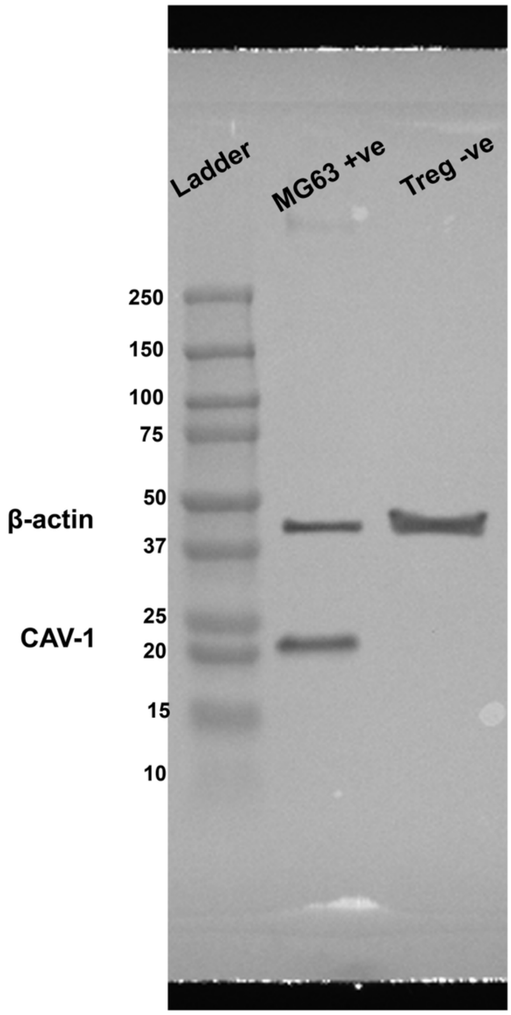

Figure S6. Western blot analysis of CAV-1 expression. *Chemiluminescence detection of β-actin and CAV-1 under identical exposure conditions, with the complete membrane displayed. Lane order (left to right): Ladder, MG63 +ve and Treg -ve.*

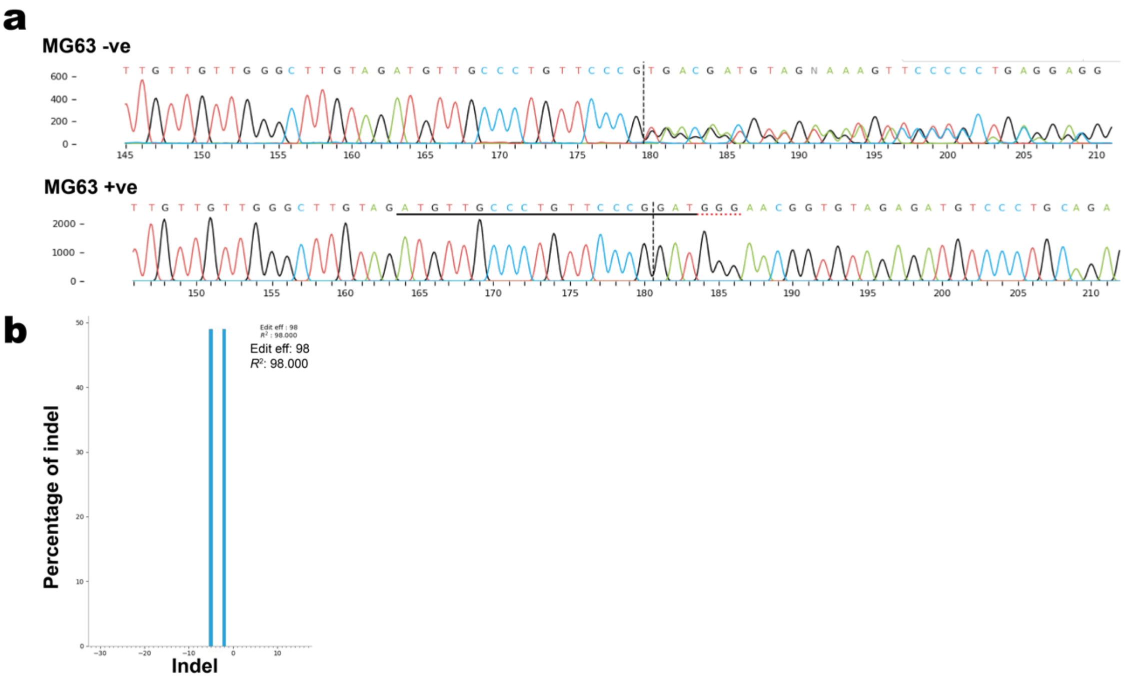

Figure S7. Validation of CAV-1 knockout in MG63 -ve cells. (a) *Sanger sequencing chromatograms showing the successful knockout of CAV-1 in MG63 -ve Clone2 (MG63 -ve C2) compared to MG63 +ve cells. MG63 -ve C2 was used for further experiments.* ***(b)*** *Quantification of indels for MG63 -ve C2. Gene editing efficiency was estimated at 98%.*

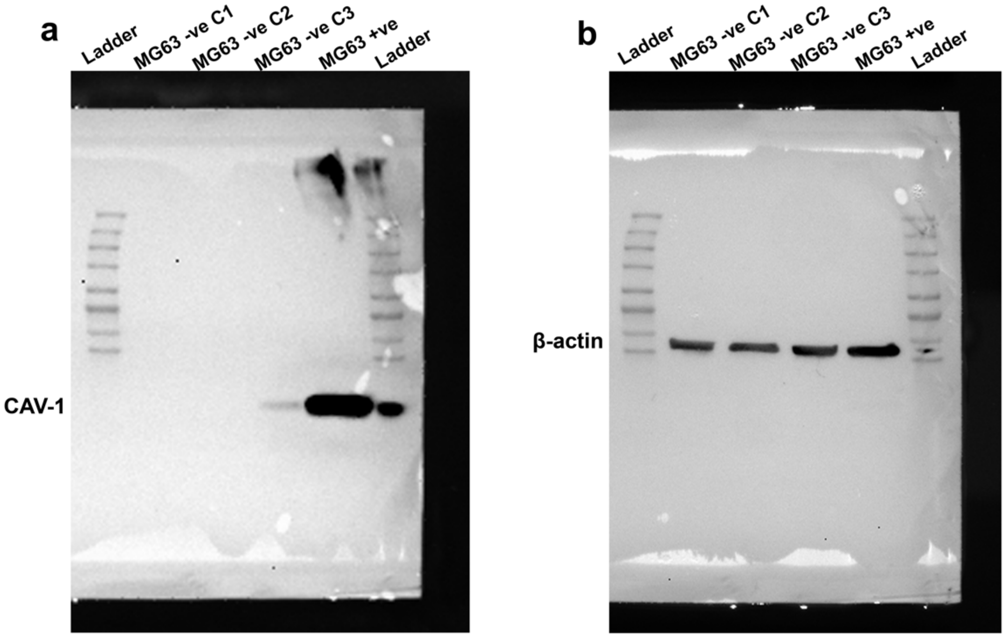

Figure S8. Western blot analysis of CAV-1 expression. (a) *Chemiluminescence detection of CAV-1 in MG63 -ve clones following CAV-1 knockout. MG63 +ve was used as a positive control.*

Lane order (left to right): Ladder, MG63 -ve C1, MG63 -ve C2, MG63 -ve C3, MG63 +ve and Ladder. ***(b)*** *Chemiluminescence detection of β-actin on the same membrane shown in (a).*

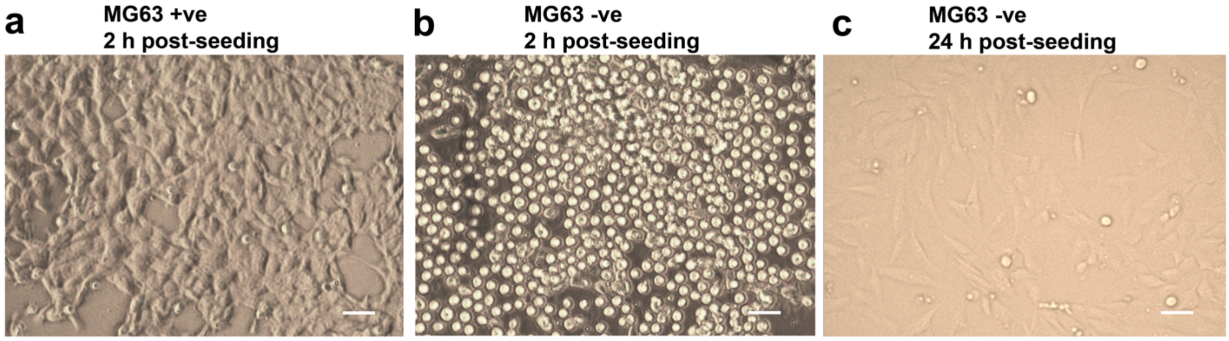

Figure S9. Morphology of MG63 +ve and MG63 -ve cells. (a) *MG63 +ve cells adhering and spreading at 2 h post-seeding in a well plate.* ***(b)*** *Loss of CAV-1 reduced adhesion and spreading within 2 h post-seeding in MG63 -ve cells.* ***(c)*** *MG63 -ve cells displayed a morphology comparable to MG63 +ve at later time point, 24 h post-seeding. Scale bar: 10 μm.*

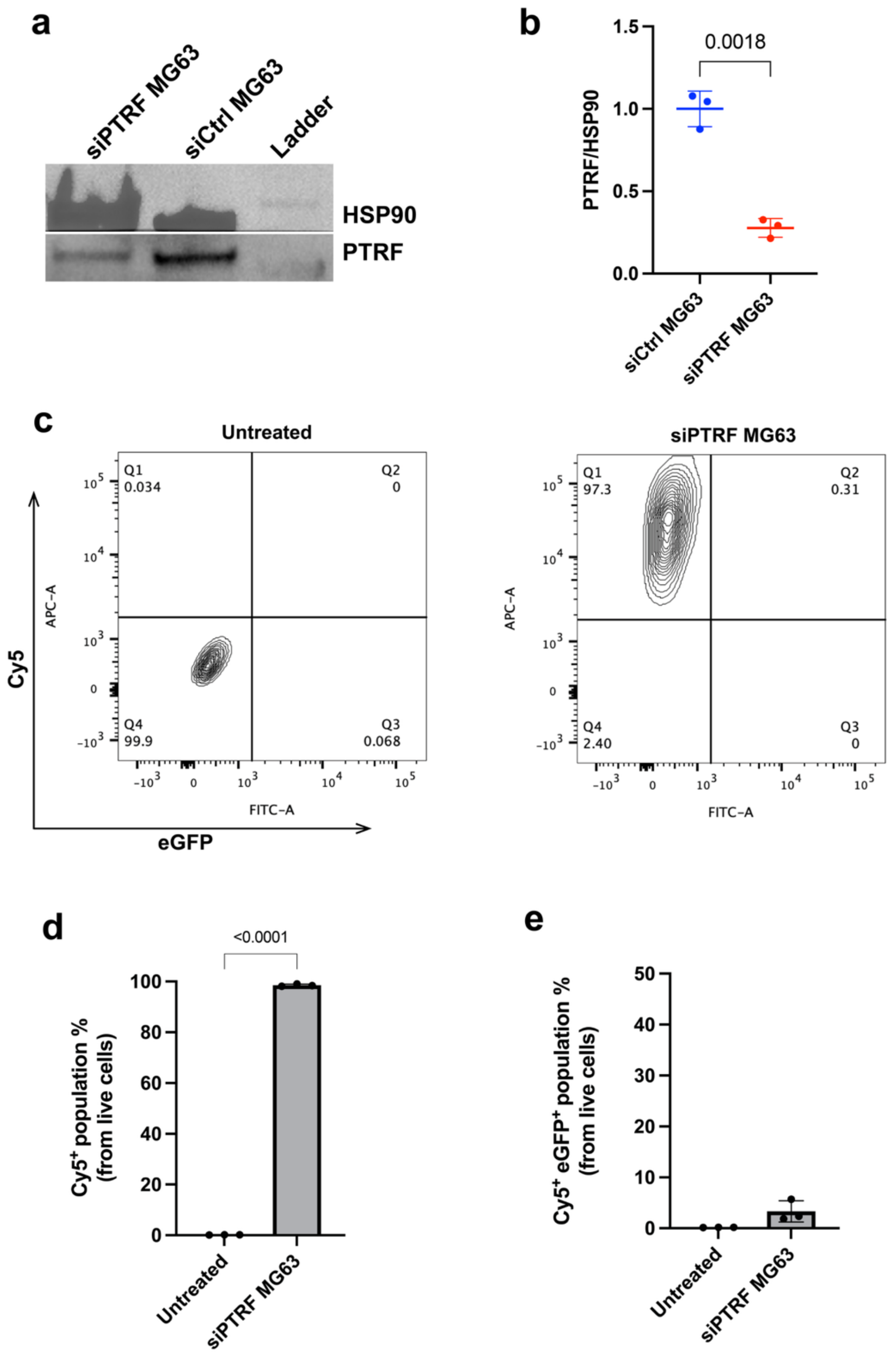

Figure S10. Validation of PTRF silencing and its impact on nanoneedle-mediated transfection in MG63 cells. (a) *Western blot showing markedly decreased PTRF protein levels in siPTRF MG63 compared with siCtrl MG63. HSP90 served as the loading control.* ***(b)*** *Quantification of PTRF reduction following siPTRF transfection. Data presented as mean ± SD, n = 3 independent samples, unpaired t test, p-value is indicated above the bar.* ***(c)*** *Representative flow cytometry contour plots of the delivery (Y-axis, Cy5 fluorescence) and transfection (X-axis, eGFP fluorescence) of Cy5-eGFP in untreated and siPTRF MG63 post nanoinjection.* ***(d)*** *Quantification of delivery efficiency (Cy5$^+$ cell population: Q1 + Q2) in untreated and siPTRF MG63 following*

nanoinjection. Data presented as mean ± SD, n = 3 independent samples, unpaired t test. p-value is indicated above the bar. ***(e)*** *Quantification of transfection efficiency (Cy5⁺ eGFP⁺ cell population: Q2) in untreated and siPTRF MG63 following nanoinjection. Data presented as mean ± SD, n = 3 independent samples, unpaired t test.*

a

b

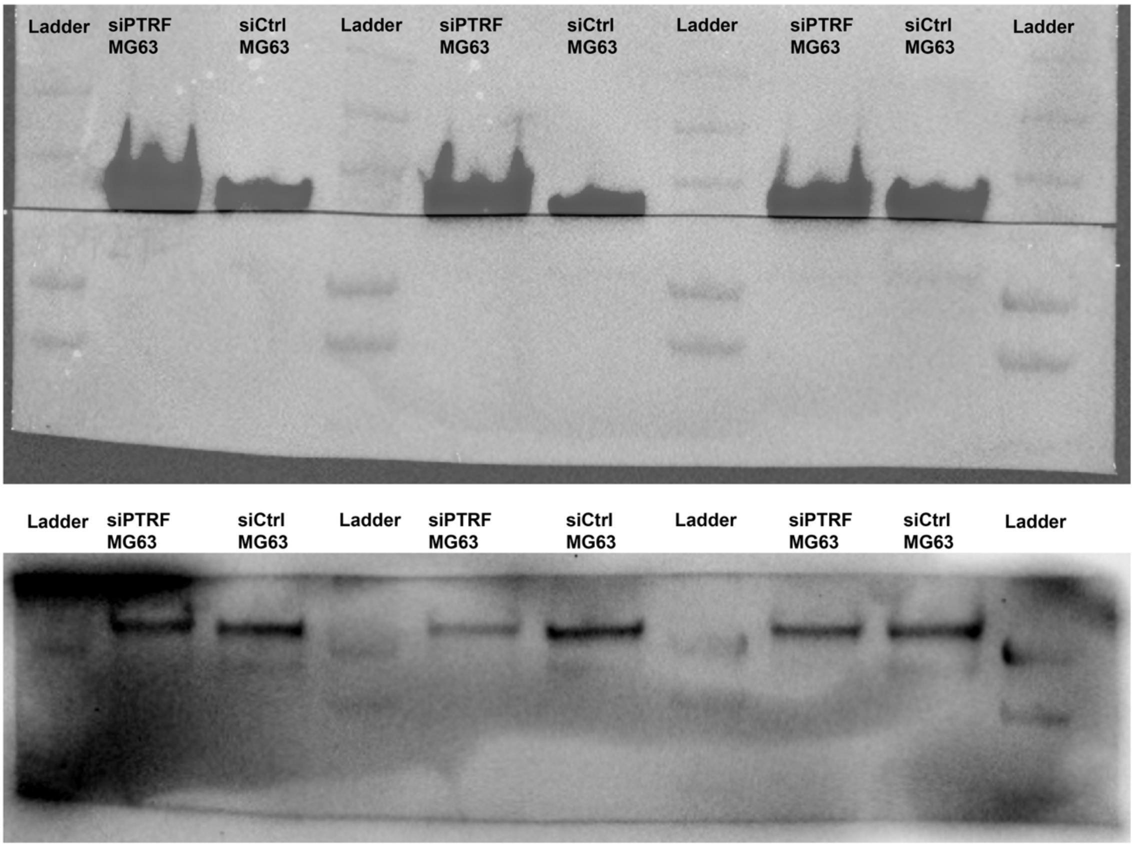

Figure S11. Western blot analysis from three independent biological replicates used for PTRF quantification in Figure S10 b. (a) *Chemiluminescence detection of HSP90, shown as a loading control, with the complete membrane displayed and a demarcation line indicating the separation between the HSP90 and PTRF regions. Lane order (left to right): Ladder, siPTRF MG63 (Rep1), siCtrl MG63 (Rep1), Ladder, siPTRF MG63 (Rep2), siCtrl MG63 (Rep2), Ladder, siPTRF MG63 (Rep3), siCtrl MG63 (Rep3), Ladder.* ***(b)*** *Chemiluminescence detection of PTRF on the corresponding membrane, presented individually for clarity.*

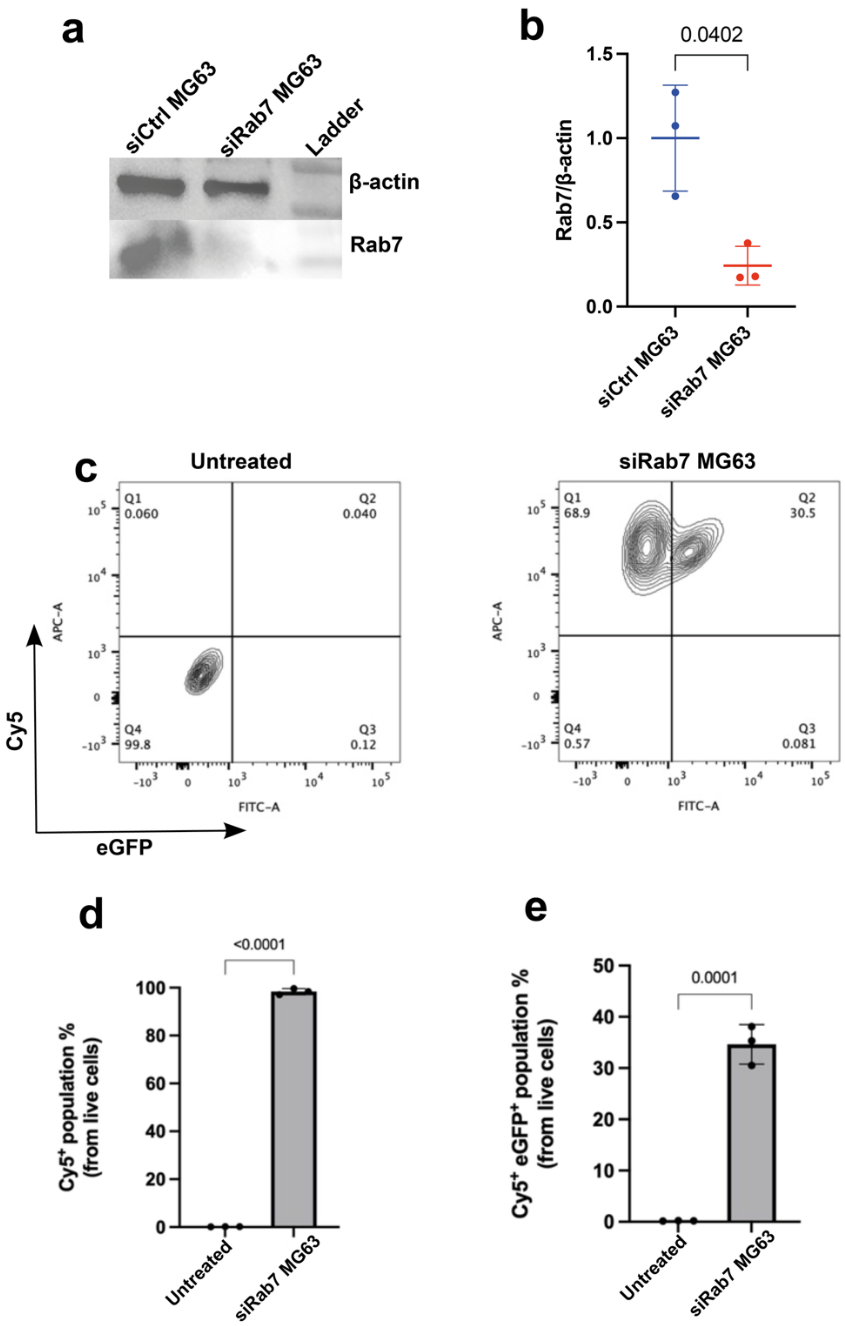

Figure S12. Validation of Rab7 silencing and its impact on nanoneedle-mediated transfection in MG63 cells. (a) *Western blot showing markedly decrease Rab7 protein levels in siRab7 MG63 compared with siCtrl MG63. β-actin served as the loading control.* ***(b)*** *Quantification of Rab7 reduction following siRab7 transfection. Data presented as mean ± SD, n = 3 independent samples, unpaired t test, p-value is indicated above the bar.* ***(c)*** *Representative flow cytometry contour plot of the delivery (Y-axis, Cy5 fluorescence) and transfection (X-axis, eGFP fluorescence) of Cy5-eGFP in untreated and siRab7 MG63 post nanoinjection.* ***(d)*** *Delivery efficiency ($Cy5^+$cell population: Q1 + Q2) in untreated and siRab7 MG63 post nanoinjection. Data*

presented as mean ± SD, n = 3 independent samples, unpaired t test. p-value is indicated above the bar. ***(e)*** *Transfection efficiency (Cy5^{+} eGFP^{+} cell population: Q2) in untreated and siRab7 MG63 post nanoinjection. Data presented as mean ± SD, n = 3 independent samples, unpaired t test, p-value is indicated above the bar.*

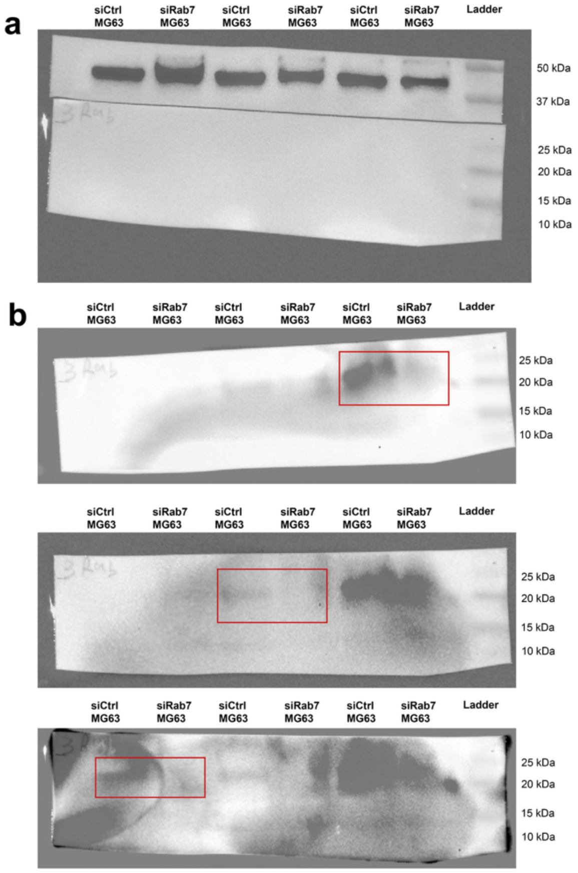

Figure S13. Western blot analysis from three independent biological replicates used for quantification shown in Figure S12 b. (a) *Chemiluminescence detection of β-actin, shown as a loading control, with the complete membrane displayed and a demarcation line indicating the separation between the β-actin and Rab7 regions. Lane order (left to right): siCtrl MG63 (Rep1), siRab7 MG63 (Rep1), siCtrl MG63 (Rep2), siRab7 MG63 (Rep2), siCtrl MG63 (Rep3), siRab7 MG63 (Rep3), and Ladder.* ***(b)*** *Chemiluminescence detection of Rab7 under three different exposure settings for the triplicate loading samples (labelled as red square) in the same membrane.*

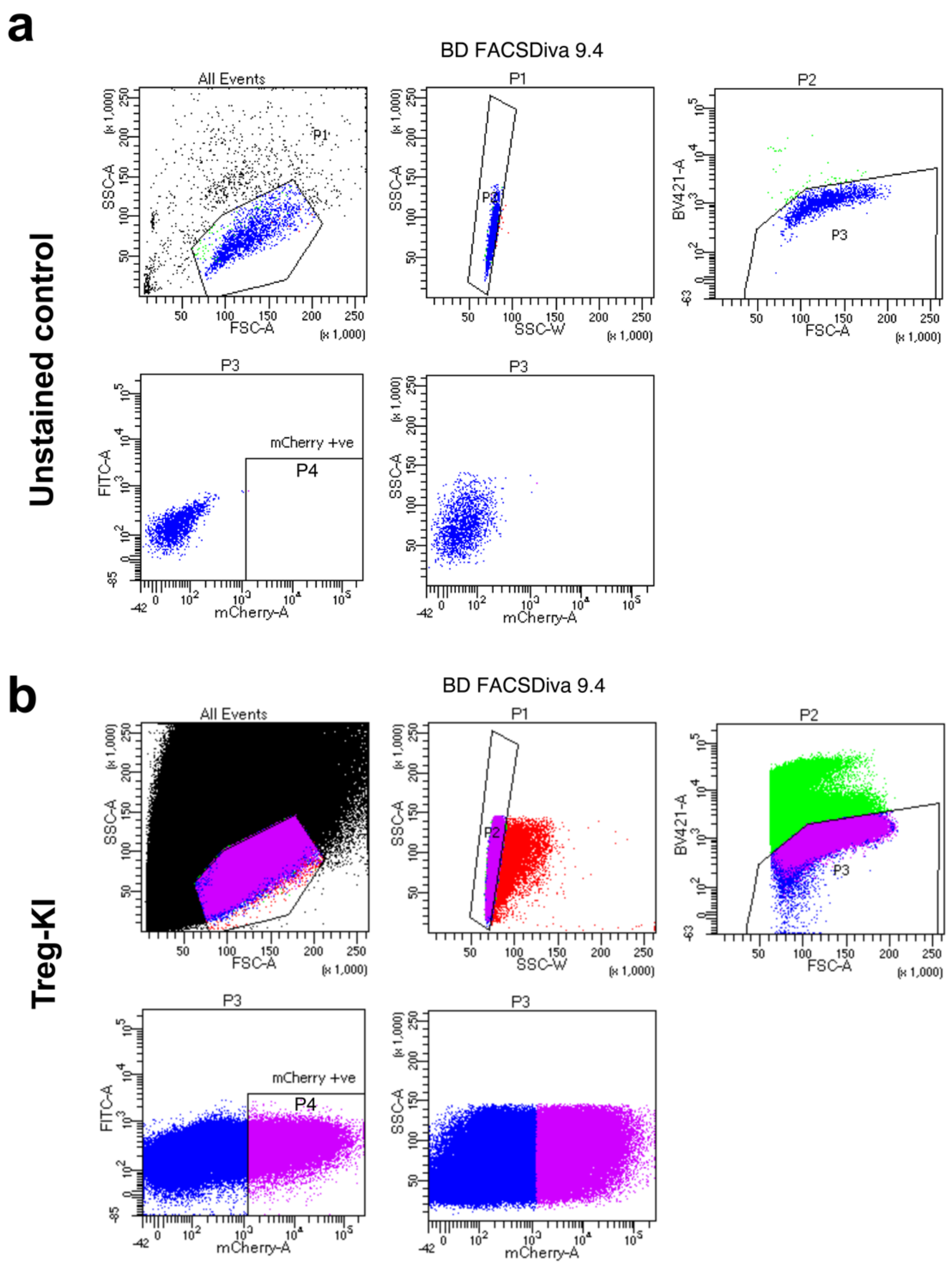

Figure S14. Representative gating strategy of Treg +ve cell sorted from Treg-KI population. *Unstained control served as negative control. P1: Lymphocytes from all events; P2: Single cell population from P1; P3: Selected part of viable cell population (BV421 -ve) from P2; P4: CAV-1 positive cell population (mCherry +ve) from P3.*

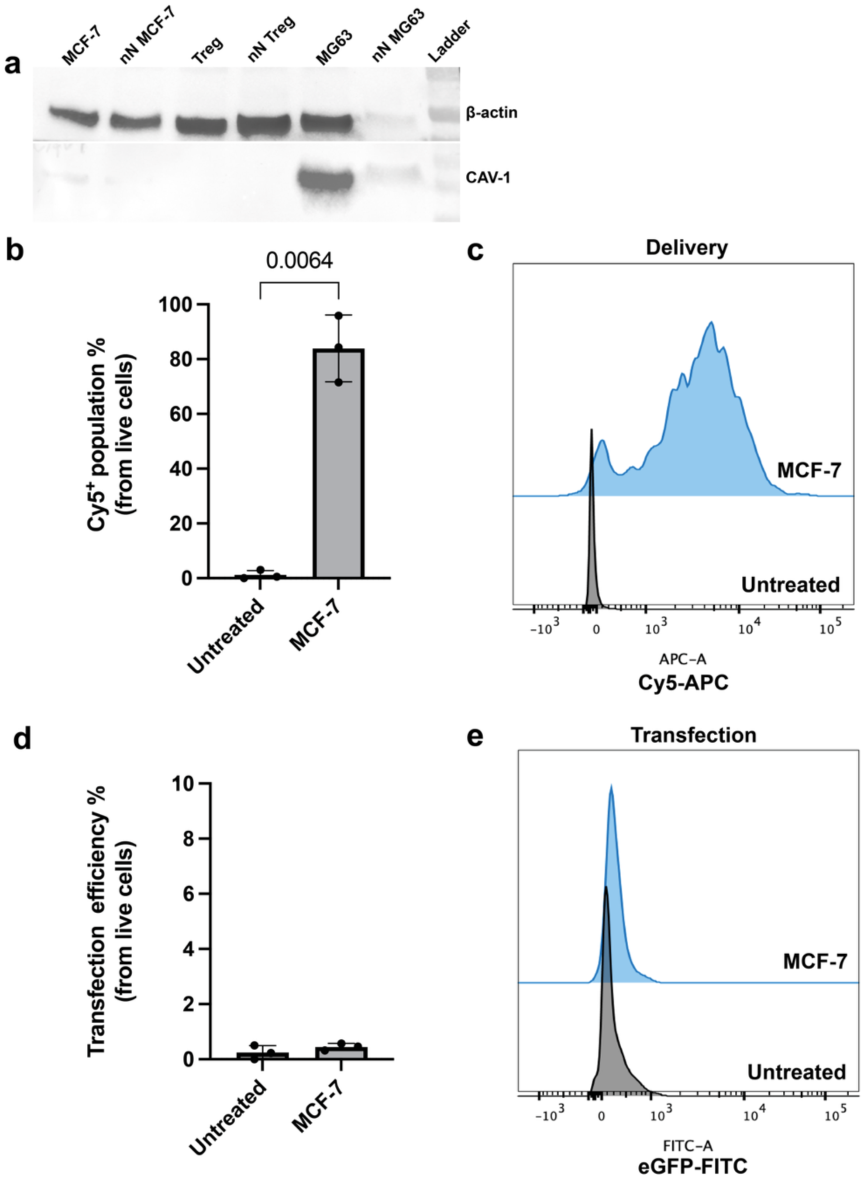

Figure S15. Transfection efficiency in MCF-7. (a) *Western blot validation of low CAV-1 expression level in MCF-7 with or without nanoinjection. β-actin was used as a loading control. Tregs and MG63 served as negative and positive controls respectively.* ***(b)*** *Delivery efficiency ($Cy5^+$cell population) in untreated and MCF-7 cells post nanoinjection. Data presented as mean ± SD, n = 3 independent samples, unpaired t test. p-value is indicated above the bar.* ***(c)*** *Representative flow cytometry histogram of the Cy5 fluorescence in untreated and MCF-7 cells post nanoinjection.* ***(d)*** *Transfection efficiency ($Cy5^+$ $eGFP^+$ cell population) in untreated and MCF-7 cells post nanoinjection. Data presented as mean ± SD, n = 3 independent samples, unpaired t test.* ***(e)*** *Representative flow cytometry histogram of the eGFP fluorescence in untreated and MCF-7 cells post nanoinjection.*

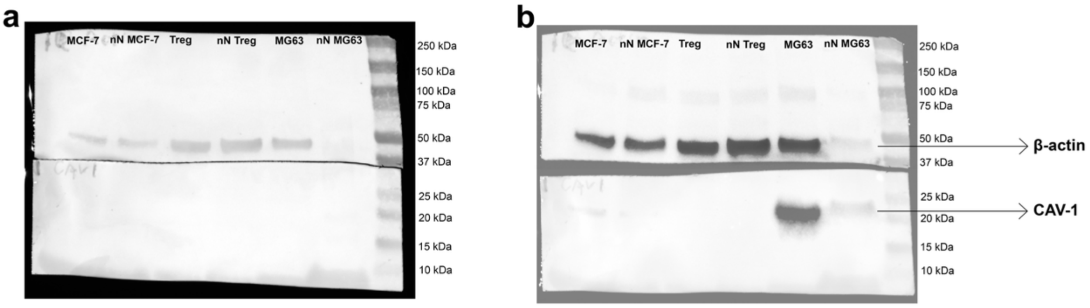

Figure S16. Western blot analysis shown in Figure S15 a. (a) *Colorimetric blot image corresponding to Figure S15 a, with the complete membrane displayed and a demarcation line indicating the separation between β-actin and CAV-1 regions.* ***(b)*** *Chemiluminescence detection of β-actin and CAV-1 under identical exposures conditions.*
